# Functional alterations by a subgroup of neonicotinoid pesticides in human dopaminergic neurons

**DOI:** 10.1007/s00204-021-03031-1

**Published:** 2021-03-29

**Authors:** Dominik Loser, Maria G. Hinojosa, Jonathan Blum, Jasmin Schaefer, Markus Brüll, Ylva Johansson, Ilinca Suciu, Karin Grillberger, Timm Danker, Clemens Möller, Iain Gardner, Gerhard F. Ecker, Susanne H. Bennekou, Anna Forsby, Udo Kraushaar, Marcel Leist

**Affiliations:** 1 NMI Natural and Medical Sciences Institute at the University of Tübingen, 72770 Reutlingen, Germany; 2 NMI TT GmbH, 72770 Reutlingen, Germany; 3 In Vitro Toxicology and Biomedicine, Department Inaugurated by the Doerenkamp-Zbinden Foundation, University of Konstanz, Universitaetsstr. 10, 78457 Konstanz, Germany; 4 Life Sciences Faculty, Albstadt-Sigmaringen University, 72488 Sigmaringen, Germany; 5 Department of Biochemistry and Biophysics, Stockholm University, 106 91 Stockholm, Sweden; 6 Department of Pharmaceutical Sciences, University of Vienna, Vienna, Austria; 7 CERTARA UK Limited, Simcyp Division, Level 2-Acero, 1 Concourse Way, Sheffield, S1 2BJ UK; 8 Technical University of Denmark, Kongens Lyngby, Denmark

**Keywords:** Live-cell calcium imaging, Neurotoxicity, Nicotine, Desensitization, Molecular docking

## Abstract

Neonicotinoid pesticides, originally developed to target the insect nervous system, have been reported to interact with human receptors and to activate rodent neurons. Therefore, we evaluated in how far these compounds may trigger signaling in human neurons, and thus, affect the human adult or developing nervous system. We used SH-SY5Y neuroblastoma cells as established model of nicotinic acetylcholine receptor (nAChR) signaling. In parallel, we profiled dopaminergic neurons, generated from LUHMES neuronal precursor cells, as novel system to study nAChR activation in human post-mitotic neurons. Changes of the free intracellular Ca^2+^ concentration ([Ca^2+^]_i_) were used as readout, and key findings were confirmed by patch clamp recordings. Nicotine triggered typical neuronal signaling responses that were blocked by antagonists, such as tubocurarine and mecamylamine. Pharmacological approaches suggested a functional expression of α7 and non-α7 nAChRs on LUHMES cells. In this novel test system, the neonicotinoids acetamiprid, imidacloprid, clothianidin and thiacloprid, but not thiamethoxam and dinotefuran, triggered [Ca^2+^]_i_ signaling at 10–100 µM. Strong synergy of the active neonicotinoids (at low micromolar concentrations) with the α7 nAChR-positive allosteric modulator PNU-120596 was observed in LUHMES and SH-SY5Y cells, and specific antagonists fully inhibited such signaling. To provide a third line of evidence for neonicotinoid signaling via nAChR, we studied cross-desensitization: pretreatment of LUHMES and SH-SY5Y cells with active neonicotinoids (at 1–10 µM) blunted the signaling response of nicotine. The pesticides (at 3–30 µM) also blunted the response to the non-α7 agonist ABT 594 in LUHMES cells. These data show that human neuronal cells are functionally affected by low micromolar concentrations of several neonicotinoids. An effect of such signals on nervous system development is a toxicological concern.

**Supplementary Information:**

The online version contains supplementary material available at 10.1007/s00204-021-03031-1.

## Introduction

Neonicotinoids are a class of insecticides that trigger nervous system disturbances by activation of the nicotinic acetylcholine (ACh) receptor (nAChR) (Brown et al. 2006; Tan et al. 2007). The compounds are widely used in agriculture for pest control (Jeschke et al. 2011; Goulson 2013; Casida and Durkin 2013), and they have been optimized to display a high species specificity. In general, they show a high potency on insect receptors, while they have been designed for low affinities on mammalian receptors (Tomizawa et al. 2000; Tomizawa and Casida 2003, 2005; Casida 2018). In regulatory studies, they have been proven to be relatively well-tolerated by rats, but some studies indicate an impact of neonicotinoids on mammals (Abou-Donia et al. 2008; Duzguner and Erdogan 2012; Gibbons et al. 2015; Burke et al. 2018; Berheim et al. 2019; Thompson et al. 2020).

Some doubts on the extent of the species selectivity have been raised by experiments on neonatal rat neurons, where acetamiprid and imidacloprid triggered signaling effects at concentrations as low as 1–10 µM (Kimura-Kuroda et al. 2012). As nicotine signaling can affect the development of the mammalian nervous system as typical developmental neurotoxicant (Levin et al. 1993; LeSage et al. 2006; Aschner et al. 2017), concerns have been voiced that exposure of humans to neonicotinoids may bear the risk of developmental neurotoxicity (DNT). Clarification of neonicotinoid signaling in human neurons is, therefore, of high toxicological interest (EFSA Panel on Plant Protection Products and their Residues (PPR) 2013).

The endogenous neurotransmitter ACh affects neuronal activity via the activation of (so-called muscarinic) metabotropic receptors (mAChR) and (so-called nicotinic) ionotropic nAChR. The latter are a class of homo- or heteromeric ligand-gated channels with variable permeability for Na^+^ and/or Ca^2+^ (Fucile 2004). Their activation leads to cell depolarization, to the activation of voltage-dependent Ca^2+^ channels, and thus, to an increase of the intracellular free Ca^2+^ concentration ([Ca^2+^]_i_). A large number of genes coding for subunits of nAChRs have been identified, and all functional receptors are pentamers formed from either a single subunit (e.g., α7), two subunits (e.g., α4 and β2) or > 2 subunits. The subunit composition determines the ligand affinities and the gating properties (Nelson et al. 2003; Mihalak et al. 2006; Jonsson et al. 2006; Carbone et al. 2009; Capelli et al. 2011). For instance, α7 receptors exhibit fast inactivating kinetics (Elliott et al. 1996; Mihalak et al. 2006; Alijevic et al. 2020), while, e.g., β2-containing receptors show slower inactivation properties (Elliott et al. 1996; Nelson et al. 2003; Mihalak et al. 2006; Alijevic et al. 2020). Both types of receptors are expressed in the central nervous system. E.g., nAChRs are expressed on dopaminergic neurons, where they regulate the release of dopamine (Rapier et al. 1988; Grady et al. 1992; Quik and Kulak 2002; Mameli-Engvall et al. 2006; Quik and Wonnacott 2011; de Kloet et al. 2015).

Substances that alter neurotransmitter signaling transiently (while they are present) in the adult nervous system may have long-term consequences (even for years after their actual exposure) in the developing nervous system (Slikker Jr et al. 2005; Grandjean and Landrigan 2006, 2014; Smirnova et al. 2014). This is not only due to the often-cited high vulnerability of the developing brain (Rice and Barone Jr 2000; Fritsche et al. 2018), but rather related to the fact that neurotransmitter signaling takes different roles in the developing and adult nervous system (Nguyen et al. 2001). While the brain is formed, GABA does not only serve short-term communication, but it also triggers synaptogenesis (Oh et al. 2016), synapse pruning (Wu et al. 2012), neuronal migration (Li et al. 2018), stem cell fate (Song et al. 2012) and neurogenesis (Kriegstein 2005; Tozuka et al. 2005). Other neurotransmitters take similar roles, such as spine growth triggered by glutamate (Kwon and Sabatini 2011), and various neurogenic processes affected by serotonin (Schaefer et al. 2013; Migliarini et al. 2013; Agrawal et al. 2019), or nicotine (Slikker Jr et al. 2005; Dwyer et al. 2009; Slotkin et al. 2016; Zahedi et al. 2019). The toxicological consequence is that disturbance of neurotransmitter signaling during development can result in an altered brain connectivity in later life. Therefore, compounds showing acute interferences with adult neurotransmission need to be considered as potential developmental neurotoxicants (Grandjean and Landrigan 2006, 2014; Smirnova et al. 2014).

Hundreds of signaling molecules and alterations in the nervous system converge to very few cellular changes, such as alterations of the neuronal membrane potential and modulation of ion channels. A traditional method to assess this is to pre-load cells with radioactive ^86^Rb^+^ and to quantify the efflux upon stimulation. Using this method, SH-SY5Y neuroblastoma cells have been established as model to study nAChR signaling (Lukas et al. 1993; Tomizawa and Casida 1999). Cell membrane depolarization activates voltage-dependent Ca^2+^ channels, leading to a transient increase of the [Ca^2+^]_i_, and some nAChRs in neurons are directly permeable for Ca^2+^ (reviewed by Fucile 2004). Therefore, Ca^2+^-imaging can be used to capture many different types of signaling events. The detection can be performed at high throughput using live-cell fluorescence imaging of neuronal cultures loaded with calcium-sensitive dyes (Leist and Nicotera 1998; Sirenko et al. 2019; Grunwald et al. 2019; Karreman et al. 2020; Brüll et al. 2020). A more direct, but lower throughput method to assess the neuronal membrane potential is patch clamp electrophysiology. This approach can directly measure the voltage or the current flow over neuronal membranes (Smith et al. 1992; Kang and Kitai 1993; Molleman 2003; Cummins et al. 2009).

The neuronal precursor cell line LUHMES is a widely used model to study biochemical and pharmacological effects on the human nervous system, as well as various types of toxicities (Krug et al. 2013, 2014; Zhang et al. 2014; Lohren et al. 2015; Smirnova et al. 2016; Harris et al. 2017; Tong et al. 2017; Witt et al. 2017; Delp et al. 2018a, b, 2019, 2021; Brüll et al. 2020). The cells can be differentiated into post-mitotic dopaminergic neurons (Scholz et al. 2011), and we have recently shown their usefulness for functional neurotoxicity assessment, including the measurement of effects on ionotropic (purinergic) receptors (Loser et al. 2021). The SH-SY5Y neuroblastoma cells are another human model, which has been used for studies on Ca^2+^ signaling in neuronal function such as mAChR and voltage-dependent Ca^2+^ channel activation (Gustafsson et al. 2010). The cells express several nAChR subunits (Gould et al. 1992; Peng et al. 1994) and nAChRs have been the target for neurotoxicity studies (Ring et al. 2015) and investigations on the role in amyloid processing (Mousavi and Hellström-Lindahl 2009) using the SH-SY5Y cell model.

Here, we characterized functional nAChRs on LUHMES neurons, and we asked whether SH-SY5Y and LUHMES cell cultures can be utilized for the assessment of xenobiotic effects on nAChRs. After confirming that the test systems indeed delivers robust and literature-congruent data on nicotinic signaling, we evaluated the six neonicotinoids with the highest market share (Jeschke et al. 2011; Bass et al. 2015), namely acetamiprid (Aceta), imidacloprid (Imida), clothianidin (Cloth), thiacloprid (Thiac), thiamethoxam (Thiam) and dinotefuran (Dino). Our studies’ overall goal was to identify potential modulatory effects of these pesticides on human neuronal [Ca^2+^]_i_ homeostasis. As it is known that disturbed cholinergic signaling, e.g., by exposure to nicotine, may trigger developmental neurotoxicity, the underlying rationale for this study was to evaluate the propensity for neonicotinoids to replicate nicotine toxicity in preterm or neonate infants.

## Materials and methods

### Materials and chemicals

An overview of experimental tool compounds and toxicants is given in table S1. Chemical structures of acetylcholine (https://pubchem.ncbi.nlm.nih.gov/compound/187#section=2D-Structure), cytisine (https://pubchem.ncbi.nlm.nih.gov/compound/10235#section=2D-Structure), nicotine (https://pubchem.ncbi.nlm.nih.gov/compound/89594#section=2D-Structure) and varenicline (https://pubchem.ncbi.nlm.nih.gov/compound/170361#section=2D-Structure) were obtained from PubChem and visualized in ChemDraw JS (version 19.0.0-CDJS-19.0.x.9 + da9bec968, PerkinElmer).

### LUHMES cell culture

The cultivation of the LUHMES cells was performed as described earlier (Scholz et al. 2011; Krug et al. 2013; Schildknecht et al. 2013). In brief, LUHMES cells were cultured in standard cell culture flasks (Sarstedt) that were pre-coated with 50 µg/ml poly-l-ornithine (PLO) and 1 µg/ml fibronectin (Sigma-Aldrich) in H_2_O overnight at 37 °C. The cells were maintained in proliferation medium containing advanced DMEM/F12 (Gibco) with 2 mM L-glutamine (Sigma-Aldrich), 1 × N2-supplement (Gibco) and 40 ng/ml recombinant human basic fibroblast growth factor (FGF-2, R&D Systems). The cells were kept at 37 °C and 5% CO_2_ and passaged three times a week, when the culture reached a confluency of 75–90%. Cells were used until passage 18. For differentiation, cells were cultured in differentiation medium consisting of advanced DMEM/F12 (Gibco) supplemented with 2 mM l-glutamine (Sigma-Aldrich), 1 × N2-supplement (Gibco), 1 mM N6,2′-0-dibutyryl 3′,5′-cyclic adenosine monophosphate (cAMP) (Sigma-Aldrich), 1 µg/ml tetracycline (Sigma-Aldrich) and 2 ng/ml recombinant human glial cell-derived neurotrophic factor (GDNF, R&D Systems).

For Ca^2+^-imaging, the cells were pre-differentiated for 48 h in cell culture flasks, detached and plated at a density of 20,000 cells and 30,000 cells per well on 0.1% PEI-coated 384-well and 96-well plates (Greiner Bio-One), respectively. For manual patch clamp recordings, the cells were plated at a density of 750 cells/µl on 0.1% PEI-coated glass coverslips. The cells were further differentiated for another 7–8 days. 50% of the medium was exchanged every 2–3 days.

### Cell culture of SH-SY5Y cells

SH-SY5Y cells (passage 50–70) were cultured as previously described (Attoff et al. 2016) and monthly screened for mycoplasma contamination (Lonza MycoAlert Mycoplasma Detection Kit). Briefly, they were cultured in MEM supplemented with 10% fetal bovine serum (Gibco, 31330095), 1% non-essential amino acid solution (Gibco, 11140035), 2 mM l-glutamine (Gibco, 25030024), 100 μg/ml streptomycin and 100 U/ml penicillin (Gibco, 15140122). For maintenance culture, SH-SY5Y cells were seeded at 27,000 cells/cm^2^ in 75 cm^2^ cell culture flasks (Corning). The cells were passaged once a week using TrypLE Express Enzyme (Gibco). SH-SY5Y cells were differentiated into a neuronal-like phenotype by exchanging the maintenance medium with differentiation medium consisting of DMEM/F12 (Gibco, 31330095) supplemented with 1 mM l-glutamine (Gibco, 25030024), 100 μg streptomycin/ml, 100 U penicillin/ml, 1 × N2-supplement (Gibco, 17502048) and 1 µM all-trans retinoic acid (RA, Sigma, R2625) 24 h after seeding. The cells were incubated in 100% humidity at 37 °C in air with 5% CO_2_.

For determination of cell viability, 35,000 SH-SY5Y cells/well (109,375 cells/cm^2^) were seeded in maintenance culture medium in clear 96-well plates (Corning, 3599). 24 h after seeding, maintenance medium was replaced with differentiation medium and incubated for 3 days before exposure with compounds. Cell viability was determined after 24-h exposure with nicotine or neonicotinoids by the conversion of resazurin (Sigma, R2625) to resorufin in metabolically active cells (O’Brien et al. 2000). A 20 × resazurin stock solution was prepared by dissolving 11 mg resazurin salt in 1 ml 0.1 M NaOH and adjusting to 50 ml with PBS^−/−^ (pH set to 7.8 with 0.1 M HCl). The 20 × resazurin stock solution was sterile filtered and stored at 4 °C protected from light. After exposure with compounds, 150 μl medium was removed leaving 50 μl in each well and subsequently, 50 μl of 2 × resazurin solution dissolved in DMEM/F12 was added. The plate was incubated for 3 h at 37 °C with 5% CO_2_. Resorufin fluorescence was measured at excitation 540 nm and at emission 590 nm using a FlexStation II fluorometer (Molecular Devices).

### Gene expression profiling

Five biological replicates were generated from LUHMES cells differentiated for 2, 3, 5, 6, 8, 10, and 11 days, as well as from undifferentiated LUHMES cells (day 0). Samples were analyzed via the TempO-Seq assay, which is a targeted RNA-sequencing method developed by BioSpyder Technologies Inc. (Carlsbad, CA, USA). The method is described in detail in House et al. (2017). For sample preparation, LUHMES grown in 96-well plates were lysed in 25 µl 1 × BioSpyder lysis buffer according to the manufacturer’s instructions. The lysate from 10 wells was pooled for each sample. Samples were stored at − 80 °C before shipping on dry ice to BioClavis (BioClavis, ltd., Glasgow, UK) for TempO-Seq analysis. The resulting FASTQ files were aligned using the STAR algorithm to a pseudo-transcriptome by BioClavis and eventually normalized and standardized to a data format of x gene specific counts per 1 million reads. Traditional whole-genome RNA-sequencing (RNAseq) was performed for comparison and validation. Cells were cultured in 6-well plates. For sample preparation, medium was removed and cells were lysed in TriFast reagent (Peqlab, VWR, USA). The lysate of six wells was pooled for each sample. Samples were stored at − 20 °C until they were sent on dry ice to the department of toxicogenomics at the University Maastricht, Netherlands, for RNAseq analysis.

Changes in SH-SY5Y nAChR subunit mRNA expression after exposure with nicotine or neonicotinoids were analyzed by whole-genome TempO-Seq by BioClavis (BioClavis, ltd., Glasgow, UK) as described for the LUHMES cells. Cells (35,000 cells/well) were plated in 96-well plates (Corning) and differentiated for 3 days as described above. After 6 and 24 h of exposure with nicotine and neonicotinoids, 50 µl lysis buffer (BioClavis) was added per well according to the manufacturer’s instructions, and the plates were stored at − 80 °C. Before shipment, the plates were thawed on ice and two identical samples/situations were pooled to give 100 µl in one well in a new 96-well plate kept on ice. The plate with pooled samples was stored at − 80 °C until shipment for TempO-Seq analysis.

The time course of expression in control cells was analyzed by RNA-sequencing. For this, SH-SY5Y cells were sampled at 0, 3, 6, and 9 days of differentiation. The mRNA extraction and RNA-sequencing experimental setup including data analysis has been described in Attoff et al. (2020).

### Neurite outgrowth assay

LUHMES cells were differentiated for 2 days in differentiation medium in PLO–fibronectin pre-coated cell culture flasks. On day 2 of differentiation (d2 neurons), cells were seeded at a density of 100,000 cells/cm^2^ into PLO–fibronectin pre-coated 96-well plates. After 1 h of attachment, cells were treated for 24 h with nicotine and neonicotinoids spanning a concentration range of 5 nM–100 µM. Cells were stained with H-33342 (1 µg/ml) and calcein-AM (1 µM) and high-content imaging was performed. Live cells and neurite area were assessed in parallel using an automated algorithm as described previously (Stiegler et al. 2011; Krug et al. 2013).

### ***LUHMES Ca***^***2***+^***-imaging***

Ca^2+^-imaging was performed using HT Functional Drug-Screening System FDSS/µCELL (Hamamatsu Photonics) at nominal 37 °C. The FDSS/µCell system enables the indirect recording of changes of [Ca^2+^]_i_ via a Ca^2+^-sensitive fluorescent dye. The fluorescence signal of a complete 384-well plate is acquired at once with a high-speed and high-sensitivity digital ImagEM X2 EM-CCD camera (Electron Multiplying Charge-Coupled Device, Hamamatsu Photonics), but with limited spatial resolution. Therefore, the software only determines the mean fluorescence signal of each well rather than of individual cells. For compound application, the integrated dispenser head with 384 pipette tips was used, which can add the test compound to all wells simultaneously. Cells were preincubated with Cal-520 AM (AAT Bioquest) at a concentration of 1 µM for 1 h at 37 °C. For recording, the medium was exchanged by a buffer solution containing [mM]: 135 NaCl, 5 KCl, 0.2 MgCl_2_, 2.5 CaCl_2_, 10 HEPES and 10 d-glucose, pH 7.4. Test compound application was executed after obtaining a 1.5 min baseline recording. Where applicable, a second application was executed 4.5 min after the first application. The total recording never exceeded 8 min.

For Ca^2+^-imaging experiments with a higher resolution on the single-cell level, the Cell Observer (Carl Zeiss Microscopy, GER) was used. The Ca^2+^-sensitive dye, the cell handling before the experiment and the buffer were the same as described above for the experiments with the high-throughput FDSS/µCELL system. The compounds were applied after an initial baseline recording of the fluorescence intensity of at least 10 s.

### ***Ca***^***2***+^***measurements in SH-SY5Y***

To measure acute changes in the average [Ca^2+^]_i_ of a population, SH-SY5Y cells were examined in the 96-well plate fluorescence reader FlexStation II (Molecular Devices) using the fluorophore Fura-2AM. SH-SY5Y (35,000 cells/well; 109,375 cells/cm^2^) were seeded in maintenance culture medium in black 96-well plates with clear bottom (Corning, #3603). 24 h after seeding, maintenance medium was replaced with differentiation medium. After 72 h of differentiation, Fura-2AM diluted in KRH buffer (125 mM NaCl, 5 mM KCl, 1.2 mM MgSO_4_, 1.2 mM KH_2_PO_4_, 2.0 mM CaCl_2_, 6.0 mM d-glucose, and 25 mM HEPES (free acid), pH adjusted to 7.4 by 1.0 M NaOH) was added to the medium to a final concentration of 4 µM (Gustafsson et al. 2010). The plates were incubated for 30 min at 37 °C, before cells were washed once with 200 µl KRH buffer. 90 µl of KRH buffer without or with 10 µM PNU ± 125 µM mecamylamine (Mec) was added to the Fura-2AM-loaded cells. The plate was again incubated for 20 min to allow full hydrolysis of the AM group before the experiment. The fluorescence was assessed at 37 °C in the fluorescence plate reader (FlexStation II; Molecular Devices) at two different excitation wavelengths, 340 nm for Ca^2+^-bound Fura-2 and 380 nm for free Fura-2, and at 510 nm emission every 3.1 s using bottom read settings. After 26–29 s of initial baseline recording of the fluorescence intensity, 10 µl of the compound dilution (10 times higher than the final concentration to the cells) was transferred automatically by the FlexStation II (“Flex mode”), column wise to the cell plate wells (five wells per concentration) and the fluorescence intensity was monitored for another 150 s. The ratio of fluorescence intensity at 340/380 nm was determined and the mean values from the baseline recording before addition of test compounds was set to zero. The acute change in the Ca^2+^ influx after addition of the compounds was quantified as the area under the curve using the SoftMax Pro 4.8 software (Molecular Devices). All test compounds were dissolved in DMSO and stored as 100 mM stock solutions at − 20 °C. At the day of experiments, compounds were diluted in KRH buffer in 1:3 series, with 100 µM as the highest concentration. The DMSO concentration was kept to 0.1% in all dilution steps and 0.1% DMSO in KRH buffer was also used as a negative control. KCl (30 mM) in KRH was used as a positive control. To check the implication of the α7 nAChR isoform in the cholinergic response, the α7 nAChR allosteric modulator PNU (10 µM) was used to evaluate the effect on Ca^2+^ influx triggered by nicotine and the six neonicotinoids. For desensitization studies, the cells were pre-exposed for 20 min with Thiam, Dino or Thiac (final concentration range 0.046–100 µM) together with 10 µM PNU before addition of 11 µM nicotine. The nicotine-induced Ca^2+^ influx was normalized to the Ca^2+^ response triggered by 30 mM KCl. The Ca^2+^ influx induced by the neonicotinoids was normalized to the response to 11 µM nicotine.

### Manual patch clamp recordings

Manual patch clamp experiments were performed in the whole-cell mode (Hamill et al. 1981) with an EPC 10 USB patch clamp amplifier and PatchMaster Software (version 2 × 90.5; HEKA Elektronik, Lambrecht, Germany). Extracellular solution contained [mM]: 140 NaCl, 4 KCl, 1 MgCl_2_, 1.8 CaCl_2_, 10 HEPES and 10 d-glucose, pH 7.4. Intracellular solution contained [mM]: 107 K-gluconate, 10 KCl, 1 MgCl_2_, 10 HEPES, 5 EGTA, 4 Na_2_ATP and 0.2 NaGTP, pH 7.2. Recordings were executed at room temperature. For agonist tests in current-clamp and voltage-clamp mode, cells were kept at a holding potential of − 70 mV and the compounds were applied for 5 s.

### Physicochemical properties and molecular docking studies

Based on the specific ChEMBL-ID of the substances, AlogP values as a descriptor for lipophilicity were extracted from the ChEMBL-database (https://www.ebi.ac.uk/chembl) (Davies et al. 2015; Mendez et al. 2019). The values provided by ChEMBL were calculated using RDKit (https://www.rdkit.org, 2018) based on the method described by Wildman and Crippen (Wildman and Crippen 1999). Polar surface areas (PSA values) were calculated using the maestro 2020-2 software (Schrödinger Release 2020-2 2020) suite (Ertl et al. 2000).

The cryo-EM-resolved 3D-structures for the human nAChR α4β2-subtype were extracted from the protein data bank (rcsb.org (Berman et al. 2000)). Structure PDB-ID: 6cnj was used for the α4β2-binding site because of its superior resolution compared to structure 6cnk which was used for the α4α4-binding site (Walsh et al. 2018). For the α4β2-isoform, the box center of the grid for the induced fit docking (IFD) run was chosen to be the centroid of the co-crystallized ligand nicotine between chain A and B: [A:402] in 6cnj and [A:405] in 6cnk (Walsh et al. 2018), respectively.

The structure of the human nAChR α7-isoform has not been experimentally resolved yet, but there are published homology models of the ligand-binding domain (LBD) available that were used for docking studies on this subtype (Ng et al. 2018). A recent paper from 2020 provided the information for the 3D-structure of this constructed extracellular LBD, that consists of two chains of the protein (Sakkiah et al. 2020). Previous studies characterized key ligand-binding residues on the human nAChR α7-subtype as follows (Ng et al. 2018; Sakkiah et al. 2020): Tyr32, Phe33, Ser34, Trp55, Leu56, Gln57, Met58, Ser59, Trp60, Thr77, Arg79, Trp107, Val108, Leu109, Val110, Asn111, His115, Cys116, Gln117, Tyr118, Leu119, Pro120, and Pro121 from the complementary subunit, and Ser148, Trp149, Ser150, Tyr151, Gly152, Arg186, Phe187, Tyr188, Cys190, Cys191, Lys192, Glu193, Pro194, and Tyr195 from the principal subunit. Therefore, the centroid of these amino acids around the α7-binding site was used as center of the grid for docking studies on this isoform.

Both, proteins and ligands have been prepared prior to the IFD protocol with extended sampling at pH 7 ± 0.5 using LigPrep and Protein preparation Wizard with default settings and an OPLS3e force field (Harder et al. 2016) that allowed the binding grid to adapt the residues around the ligand poses within 5 Å. The box size was set to 12 Å in Maestro (Schrödinger Release 2020-2 2020).

An induced fit docking protocol as implemented in the Schrödinger Software Suite was applied. The protocol comprises a Glide initial docking run where the ligands are docked to the previously defined grid rigidly after a constrained minimization (Prime) of the receptor has been performed (Sherman et al. 2006; Schrödinger Release 2020-2 2020). A large number of poses per ligand was generated by this first docking run, from which only a set was passed on to the next steps by applying energy filters (Sherman et al. 2006; Schrödinger Release 2020-2 2020). This was followed by Prime side-chain prediction, another Prime minimization, Glide redocking and eventually the Scoring State. The resulting poses were then used as input for interaction fingerprint clustering with the average linking method (Schrödinger Release 2020-2 2020).

### Data analysis and statistics

For the high-throughput Ca^2+^-imaging data obtained in LUHMES cells, an offset correction using the FDSS software (version 3.2) was performed. Afterwards, the data were exported and further analyzed with scripts written in R (version 3.6.3) (R Core Team 2020). The concentration–response curves were fitted using a log-logistic model described by Ritz et al. (2015), utilizing the R package *drc* with its function *drm()* and *LL2.2()* with the following equation: *f*(*x*) = *d*/[1 + exp(*b*(log(*x*) − *ẽ*))] (Ritz et al. 2015). The logarithm of the half-maximal effective concentration (logEC_50_) between 0 and the upper limit (*d*), which was set to 1 is represented by *ẽ*, *x* denotes the concentration and *b* stands for the slope parameter (Ritz et al. 2015). In cases with normalizations to responses induced by other compounds, the function *LL2.3()* was used with a variable upper limit (*d*; Ritz et al. 2015). The same equation was used to determine the half-maximal inhibitory concentration (logIC_50_). Then, the logEC_50_ and logIC_50_ values were converted into the pIC_50_ and pEC_50_ values, which are the negative logarithms to base 10.

The single-cell Ca^2+^-imaging recordings were exported and analyzed in Fiji ImageJ (version 1.52i) to get the average fluorescence signal of each cell. These signals were further analyzed in R, where a threshold detection was performed to detect responding cells. For this, the offset was corrected by subtracting the mean of 20–65% of the fluorescence signal of the pre-application period from the recording, to be robust against spontaneous activity. The threshold was defined as mean + 3 × SD of the negative control recordings, during the detection phase of 6.5 s.

The raw data of the manual patch clamp recordings were analyzed in scripts written in R. For leak subtraction, the P/4 algorithms of PatchMaster were used in voltage-clamp recordings. The following R packages were utilized for data handling: cowplot (Wilke 2019), dplyr (Wickham et al. 2020), drc (Ritz et al. 2015), ephys2 (Danker 2018), ggplot2 (Wickham 2016), htmlwidgets (Vaidyanathan et al. 2019), lemon (Edwards 2019), magick (Ooms 2020), magrittr (Bache and Wickham 2014), matrixStats (Bengtsson 2020), miniUI (Cheng 2018), modelr (Wickham 2020), multcomp (Hothorn et al. 2008), plotrix (Lemon 2006), proto (Grothendieck et al. 2016), shiny (Chang et al. 2020), shinyjs (Attali 2020), shinyTree (Trestle Technology, LLC 2017), and tidyverse (Wickham et al. 2019).

Concentration–effect responses in the SH-SY5Y [Ca^2+^]_i_ were analyzed by the GraphPad Prism8.0 software using the four-parameter sigmoidal curve fit settings and the concentrations giving 50% (BMC_50_) increase in [Ca^2+^]_i_ in relation to the nicotine response were estimated.

The raw count tables of gene expression profiling with TempO-Seq assay and traditional whole-genome RNA-sequencing (RNAseq) were analyzed with the R package DESeq2 (v1.24.0) (Love et al. 2014). RNAseq counts were normalized to the library size and the transcript length [Transcripts per kilobase million (TPM)] (Wagner et al. 2012). TempoSeq counts were normalized to total counts per sample [counts per million (CPM)]. Gene lengths were retrieved from the hg18 reference genome (NCBI Build 36.1) with the R package Goseq (v1.40.0) (Young et al. 2010). TPM/CPM were averaged over the five biological replicates.

Unless mentioned differently, values are presented as mean ± SEM. If not indicated otherwise, experiments were performed with at least three technical replicates per condition. Detailed data on pEC_50_, pIC_50_ and n numbers are found in supplementary tables. Unless mentioned differently, statistical significance was defined as *P* < 0.05 and was determined by one-way ANOVA with Dunnett’s post hoc test as indicated. To determine benchmark concentrations, and their upper and lower 95% confidence intervals (BMCL, BMCU), the BMC online software of UKN was used (Krebs et al. 2020).

## Results and discussion

### Suitability of LUHMES neurons to study human nAChR responses

**Fig. 1:**
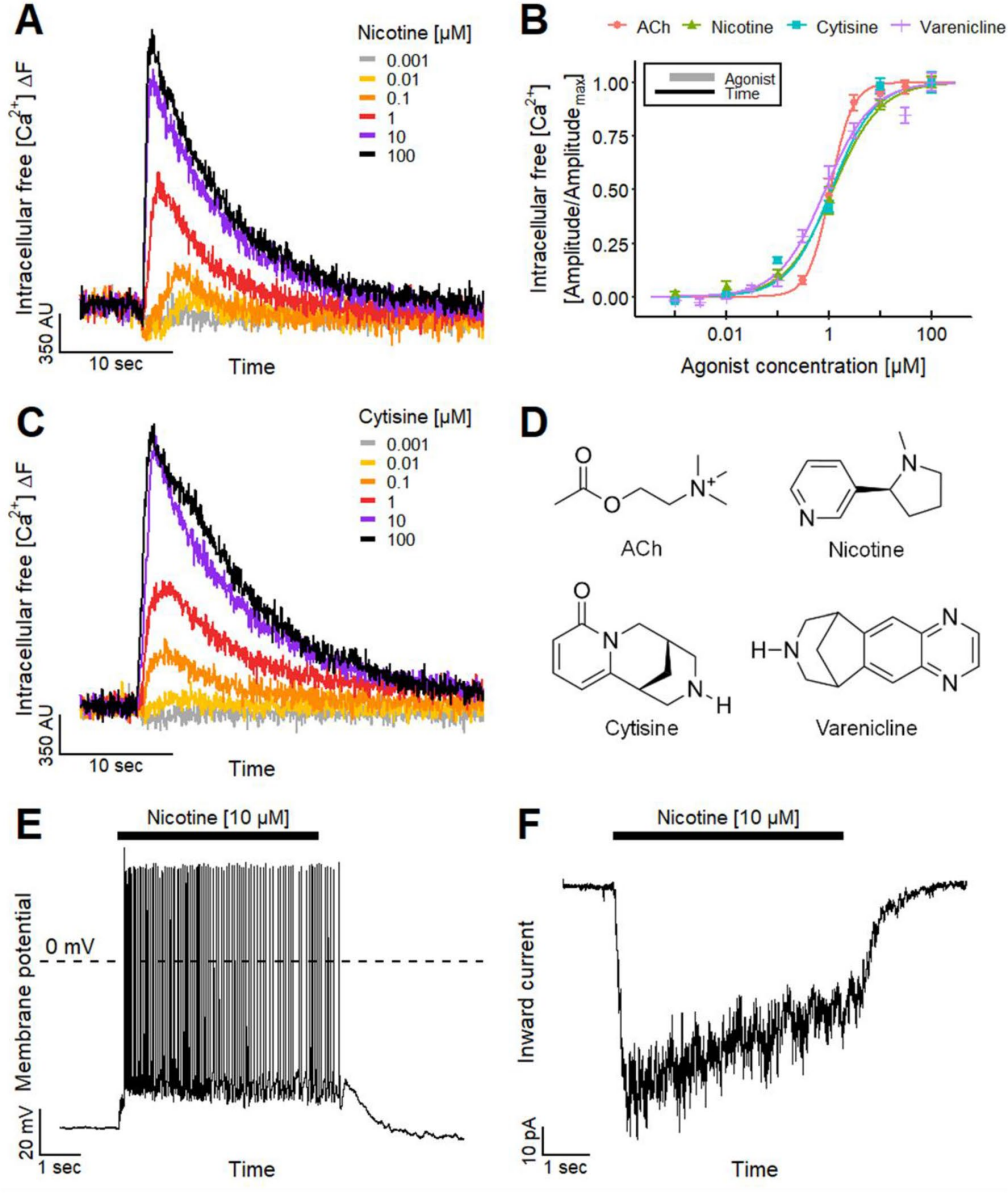
Identification of functional nicotinic acetylcholine receptors (nAChRs) on LUHMES neurons. **a** Traces of Ca^2+^-imaging recordings show the concentration-dependent effects of the nAChR agonist nicotine on LUHMES neurons. **b** Concentration–response curves for the effects of nAChR agonists acetylcholine (ACh), nicotine, cytisine and varenicline with pEC_50_ values of 5.98 ± 0.03, 5.93 ± 0.05, 5.95 ± 0.05 and 6.08 ± 0.04, respectively. Note the treatment scheme (upper left corner), illustrating the experimental design. Detailed data on n numbers are found in table S4. **c** Ca^2+^-imaging signals evoked by the addition of cytisine. **d** Chemical structures of the tested nAChR agonists. **e**, **f** Manual patch clamp recordings of the responses of LUHMES neurons evoked by the application of 10 µM nicotine for 5 s. **e** Firing of multiple action potentials with a long-lasting depolarization of the membrane potential (*n* = 28) recorded in current-clamp. **f** Slowly inactivating inward current (*n* = 12) measured in voltage-clamp

We used general gene expression profiling data to check the suitability of LUHMES as a model for an ACh signaling target cell. Two transcriptomics approaches based on RNA sequencing suggested that the cells express several subunits of the nAChR. Particularly high signals were obtained in differentiated cells for the α4, α7, and β2 chains, but also some other cholinergic components showed gene expression. For instance, ACh esterase (AChE) and the muscarinic AChR4 were up-regulated during the differentiation process (Fig. S1). Our data support the hypothesis that multiple nAChR subtypes transmit neurotransmitter signals to LUHMES cells, but gene expression data alone do not reveal information about protein expression and function. Therefore, we investigated the effect of the selective nAChR agonist nicotine by performing measurements of the [Ca^2+^]_i_ as functional endpoint. Application of nicotine resulted in a rapid and concentration-dependent transient rise in [Ca^2+^]_i_ (Fig. 1a). A quantification of the nicotine signaling yielded a pEC_50_ value of 5.9 (Fig. 1b). This finding is in line with other published datasets on human nicotinic receptors determined by other techniques, confirming the applicability of Ca^2+^-imaging used here: e.g., pEC_50_ values of 5.5–6.1 (EC_50_: 0.9–3.5 µM) have been reported for human α4β2 nAChRs using patch clamp (Buisson et al. 1996; Wu et al. 2006; Chen et al. 2018), Ca^2+^-imaging (Chavez-Noriega et al. 2000; Capelli et al. 2011) and membrane potential fluorescence (Fitch et al. 2003). Patch clamp recordings with human α6/3β2β3 (Armstrong et al. 2017; Chen et al. 2018) or α4β4 (Wu et al. 2006) nAChRs showed pEC_50_ values of ~ 5.9. In this context, it is noteworthy that muscular (non-neuronal) nAChR (α1β1δγ/ε) have clearly lower nicotine affinities (Fitch et al. 2003; Capelli et al. 2011) of around 25 µM (pEC_50_: ~ 4.6).

After these encouraging initial experiments on nicotinic signaling, we used cytisine as a second, well-established agonist. This compound exhibits high affinity and low efficacy for β2-containing nAChRs (like α4β2) (Coe et al. 2005; Rollema and Hurst 2018). The compound-induced Ca^2+^-signals were comparable in both kinetics and sensitivity to nicotine (Fig. 1c). The potency of cytisine (pEC_50_: 6.0) was also in the 1 µM range. A similar set of data (pEC_50_: 6.1) was obtained for a third nicotinic agonist, varenicline (Fig. 1b) (Mihalak et al. 2006). Again, these data were in good agreement with data obtained by several other functional test platforms (Chavez-Noriega et al. 2000; Fitch et al. 2003; Coe et al. 2005; Wu et al. 2006; Chatterjee et al. 2011; Arias et al. 2015).

As fourth agonist, we used the neurotransmitter ACh itself. The pEC_50_ of 6.0 (Fig. 1b) is in accordance with a large set of literature data on the signaling effects of this endogenous ligand (Buisson et al. 1996; Kuryatov et al. 1997; Nelson et al. 2003; Bermudez and Moroni 2006; Carbone et al. 2009; Mineur et al. 2009; Harpsøe et al. 2011; Benallegue et al. 2013; Armstrong et al. 2017). Finally, pilocarpine was used as mAChR agonist (Šantrůčková et al. 2014). Concentrations of up to 30 µM (n ≥ 7, data not shown) did not trigger any [Ca^2+^]_i_ changes. In summary, the quantitative data on four diverse nAChR agonists (Fig. 1d) indicate a functional expression of neuronal nAChRs, and the suitability of LUHMES cells to study compounds affecting the nicotinic signaling.

To investigate the electrophysiological impact on LUHMES neurons, manual patch clamp recordings were performed. Application of nicotine (10 µM) to a total of 42 neurons held in current-clamp evoked either tonic (*n* = 28, Fig. 1e) or phasic (*n* = 14, data not shown) action potential firing. Furthermore, voltage-clamp recordings revealed that cells responded with a fast-activating and slowly inactivating inward current (*n* = 12, Fig. 1f). These findings confirm the presence of functional nAChRs on LUHMES neurons as shown by Ca^2+^-imaging. Moreover, the observed long-lasting depolarization of the membrane potential over the entire application period indicates a strong contribution of non-α7 nAChRs (e.g., α4β2 or α4β4 subtypes) (Elliott et al. 1996; Wu et al. 2006; Mihalak et al. 2006; Rollema et al. 2007; Alijevic et al. 2020).

### Investigation of nAChR subtype composition in LUHMES cells

**Fig. 2:**
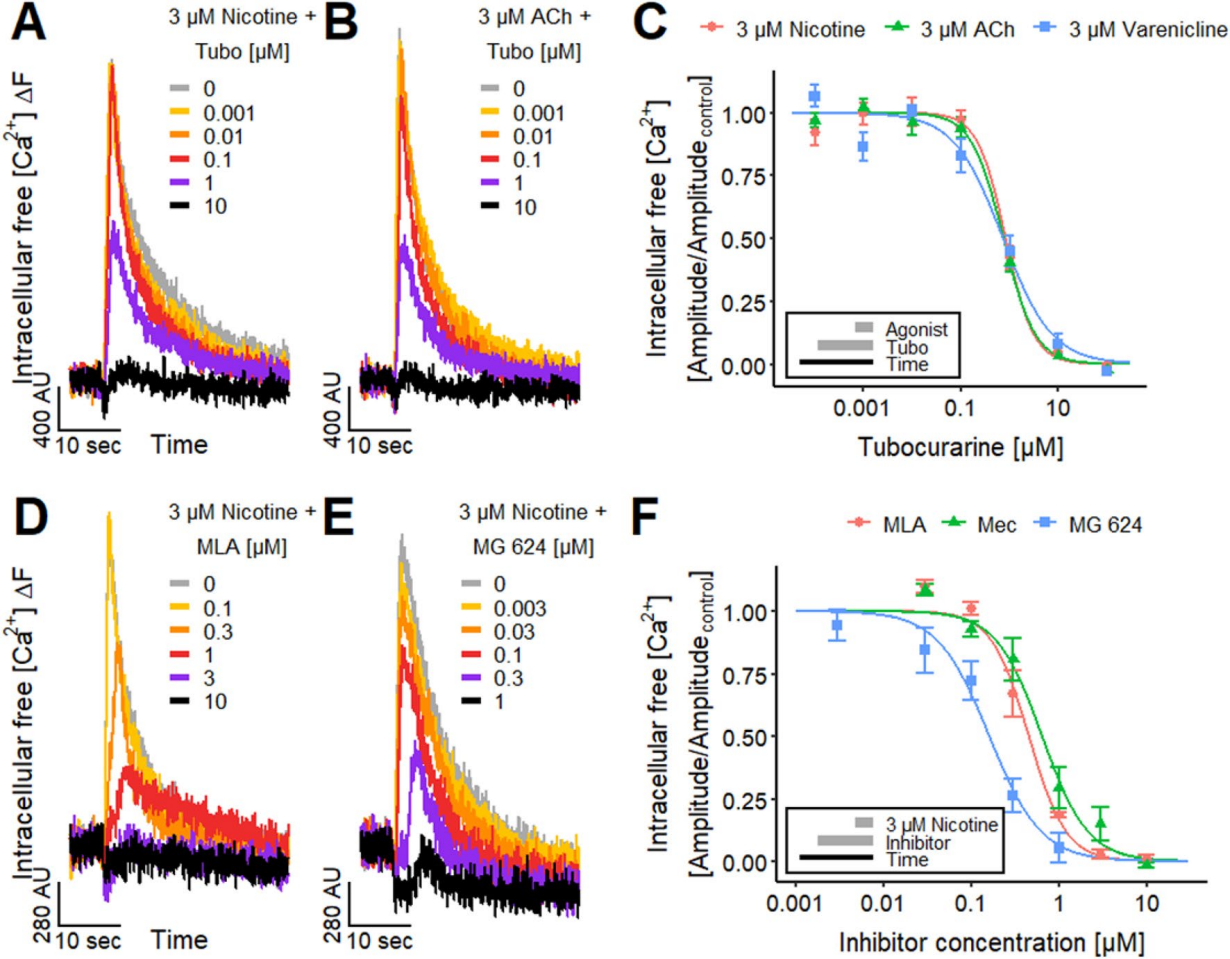
Characterization of the nAChRs. **a**, **b** Ca^2+^-imaging signals of the effects of the pre-applied non-selective nAChR antagonist tubocurarine (Tubo) on the responses of LUHMES neurons triggered by **a** 3 µM nicotine and **b** 3 µM ACh. **c** Inhibitory effect of Tubo on the signals evoked by the acute exposure to 3 µM of nicotine, ACh and varenicline. The resulting pIC_50_ values were 6.08 ± 0.04 for nicotine, 6.13 ± 0.04 for ACh and 6.13 ± 0.09 for varenicline.** d**, **e** Traces of the effects of **d** MLA and **e** MG 624 on the Ca^2+^-imaging signals stimulated by 3 µM nicotine. **f** Concentration–response curves for the effects of MLA, mecamylamine (Mec) and MG 624 on the responses triggered by the acute exposure to 3 µM nicotine. The pIC_50_ values were 6.33 ± 0.04 for MLA, 6.17 ± 0.05 for Mec and 6.80 ± 0.07 for MG 624. Note the treatment schemes (lower left corner), illustrating the experimental design. Detailed data on n numbers are found in table S4

To profile LUHMES neurons for functional neurotoxicity studies, we used additional pharmacological tools to shed light on the nAChR subtype composition. First, we tested whether antagonists of nAChRs modulated the Ca^2+^-responses. The non-selective nAChR antagonist tubocurarine (Tubo) (Jonsson et al. 2006) has a long history in toxicology and is one component of an arrow poison for hunting. Application of Tubo antagonized the responses evoked by nicotine (Fig. 2a), ACh (Fig. 2b) and varenicline concentration-dependently (pIC_50_ values ~ 6.1) (Fig. 2c). Tubo completely blocked the responses to all three agonists at concentrations > 10 µM, indicating that the entire agonist-triggered Ca^2+^-signaling was mediated by nAChRs.

To further dissect the nAChR subtypes involved in LUHMES responses, we researched the effects of other antagonists. Methyllycaconitine (MLA) is more potent (low nM range) on the α7 nAChR than on other receptor combinations (Puchacz et al. 1994; Gopalakrishnan et al. 1995; Palma et al. 1996; Buisson et al. 1996; Capelli et al. 2011). Application of MLA (Fig. 2d) yielded a pIC_50_ of 6.3 in our cell system (Fig. 2f). This low affinity indicates that mainly non-α7 nAChRs are responsible for the [Ca^2+^]_i_ response. The LUHMES pIC_50_ was similar to previously reported pIC_50_ values for human α4β2 expressed in cell lines (Buisson et al. 1996; Capelli et al. 2011), while it was different from the reported pIC_50_ values for α7, α3β4, α1β1δε and α6-containing (α6/3β2β3) nAChRs (Capelli et al. 2011). This points towards a major contribution of α4β2 to the response evoked by nicotine in LUHMES.

To further substantiate the findings resulting from the MLA experiments, we applied the non-competitive nAChR antagonist mecamylamine (Mec) (Papke et al. 2008; Capelli et al. 2011). This drug shows higher potency for α4β2 and α3β4 nAChRs compared to the a7 nAChR (Capelli et al. 2011). The pIC_50_ value of 6.2 (Fig. 2f) is comparable to literature data of ~ 6.1 for human α4β2 (Chavez-Noriega et al. 2000; Capelli et al. 2011) and 6.6 for human α3β2 (Chavez-Noriega et al. 2000), while it was different from reported pIC_50_ values for α7, α3β4, α1β1δε and α6-containing (α6/3β2β3) nAChRs (Capelli et al. 2011).

Finally, we confirmed our previous results by applying MG 624, a slightly more potent nAChR antagonist (Gotti et al. 2000; Capelli et al. 2011). The LUHMES pIC_50_ of 6.8 (Fig. 2e, f) is comparable to literature pIC_50_ values of 6.8 for α4β2, 6.6 for α3β4, 6.9 for α7 and 7.3 for α1β1δε (Capelli et al. 2011).

**Fig. 3:**
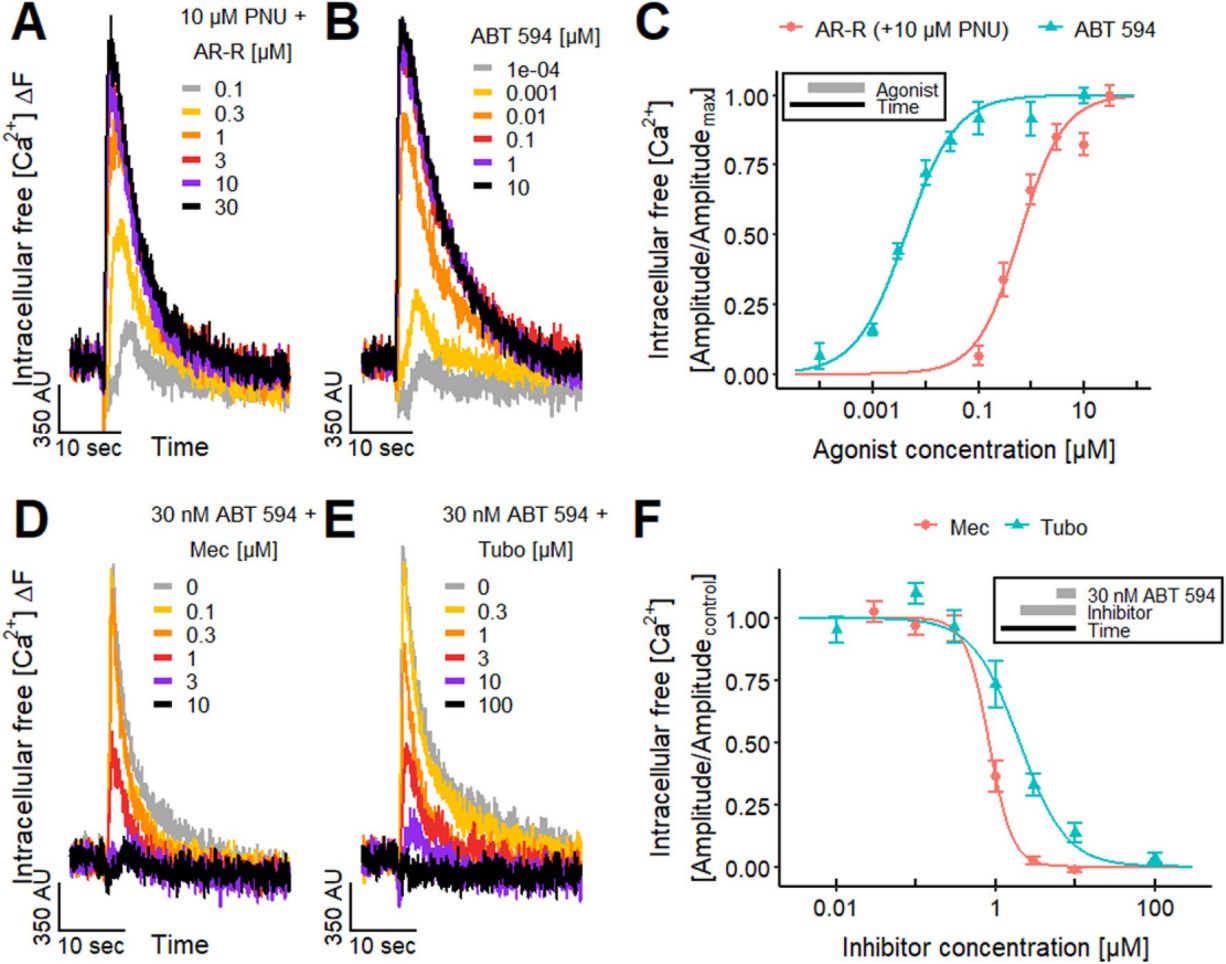
Differential agonist responses on nAChRs.** a**, **b** Signals of Ca^2+^-imaging triggered by the selective α7 nAChR agonist **a** AR-R 17779 (AR-R), after the pretreatment with 10 µM PNU-120596 (PNU), a positive allosteric modulator of α7 nAChR, and the selective non-α7 nAChR agonist **b** ABT 594. **c** Agonistic effect of AR-R in presence of 10 µM PNU and ABT 594 yielded pEC_50_ values of 6.20 ± 0.05 and 8.36 ± 0.05, respectively.** d**, **e** Ca^2+^-imaging traces of the effects of **d** Mec and **e** Tubo, which were preincubated for 4.5 min, on the response of the LUHMES neurons evoked by 30 nM ABT 594. **f** The concentration–response curves of the effects of Mec and Tubo on the response evoked by 30 nM ABT 594 resulted in pIC_50_ values of 6.08 ± 0.03 and 5.70 ± 0.05, respectively. Note the treatment schemes, illustrating the experimental design. Detailed data on n numbers are found in table S4

These antagonist data provided indirect evidence for a contribution of neuronal non-α7 nAChRs, e.g., α4β2 and/or α3-containing nAChRs, to the [Ca^2+^]_i_ responses of LUHMES evoked by nicotine. To address this issue more directly, we made use of the neuronal non-α7 nAChRs agonist ABT 594 (Donnelly-Roberts et al. 1998; Michelmore et al. 2002). We found a pEC_50_ value of 8.4 (Fig. 3b, c), which strongly suggests a presence of functional non-α7 nAChRs on LUHMES. In fact, the potency of this drug in the LUHMES system was even higher than previously reported for other cells (Donnelly-Roberts et al. 1998; Michelmore et al. 2002). This might be explained by the presence of multiple nAChR subtypes on LUHMES neurons, because the potency of ABT 594 depends on the nAChR subtype (Michelmore et al. 2002), as also described for other nAChR agonists (Chavez-Noriega et al. 2000; Jonsson et al. 2006; Capelli et al. 2011). As internal consistency check, we blocked ABT 594 responses with the nAChR antagonists Mec (Fig. 3d) and Tubo (Fig. 3e). The pIC_50_ values of 6.1 for Mec and 5.7 for Tubo (Fig. 3f) are similar to the values that we obtained before for both antagonists for nicotine (Fig. 2f).

In summary, our data demonstrate a functional expression of neuronal non-α7 nAChRs on LUHMES neurons and highlight the capability of this test system for detecting agonistic and antagonistic effects on nAChRs.

### Identification of α7 nAChR on LUHMES

As the gene expression data suggested that α7 nAChRs are highly expressed in LUHMES (Fig. S1), we investigated the functional role of this receptor subtype. First, we checked the effect of the selective α7 nAChR agonist AR-R 17779 (AR-R) (Mullen et al. 2000; Michelmore et al. 2002; Papke et al. 2004) on [Ca^2+^]_i_. We failed to detect a response (*n* ≥ 13, data not shown). The most likely reason is a very fast inactivation of the α7 nAChR (Elliott et al. 1996; Mihalak et al. 2006). This has also been described for other α7 nAChR agonists in Ca^2+^-imaging experiments (Dickinson et al. 2007; Gill et al. 2013; Chatzidaki et al. 2015; Larsen et al. 2019). As receptor desensitization can lead to technical challenges when investigating nicotinic agonists, several positive allosteric modulators including PNU-120596 (PNU) have been developed to counteract this phenomenon. We used here the selective positive allosteric modulator of the α7 nAChR, PNU-120596 (PNU). This compound can slow the α7 nAChR inactivation, and it, therefore, enables the detection of the α7 nAChR-mediated response (Hurst et al. 2005; Dickinson et al. 2007; Ng et al. 2007; Grønlien et al. 2007; Papke et al. 2009; Williams et al. 2011; Chatzidaki et al. 2015; Larsen et al. 2019). Under these conditions (presence of PNU), the cells responded concentration-dependently to the stimulation with AR-R (Fig. 3a). The pEC_50_ value of 6.2 (Fig. 3c) indicates the presence of functional α7 nAChRs. The LUHMES pEC_50_ value for AR-R is high, compared to a previously reported value for rat α7 expressed in *Xenopus laevis* oocytes (pEC_50_ = 5; Papke et al. 2004). This difference is most likely due to the increased agonist potency induced by the allosteric enhancer PNU, as previously described (Hurst et al. 2005; Grønlien et al. 2007).

To control for the α7 specificity of PNU in the LUHMES system, we tested, whether it would also enhance signaling of other receptors. In control experiments, cells were stimulated with 1 µM α,β-meATP (P2X receptor agonist (Bianchi et al. 1999; Khakh and North 2012; Loser et al. 2021)) and different concentrations of ABT 594 (non-α7 nAChR agonist). In both cases, no differences between recordings with and without PNU were detected (Fig. S2). We, therefore, conclude that PNU did not enhance [Ca^2+^]_i_ responses in general, but only those of the α7 nAChR.

In summary, the α7 nAChR-selective tool compounds showed consistent responses and interactions, thereby showing functional expression of α7 nAChRs on LUHMES neurons.

### Direct effect of neonicotinoids on otherwise untreated LUHMES cultures

**Fig. 4:**
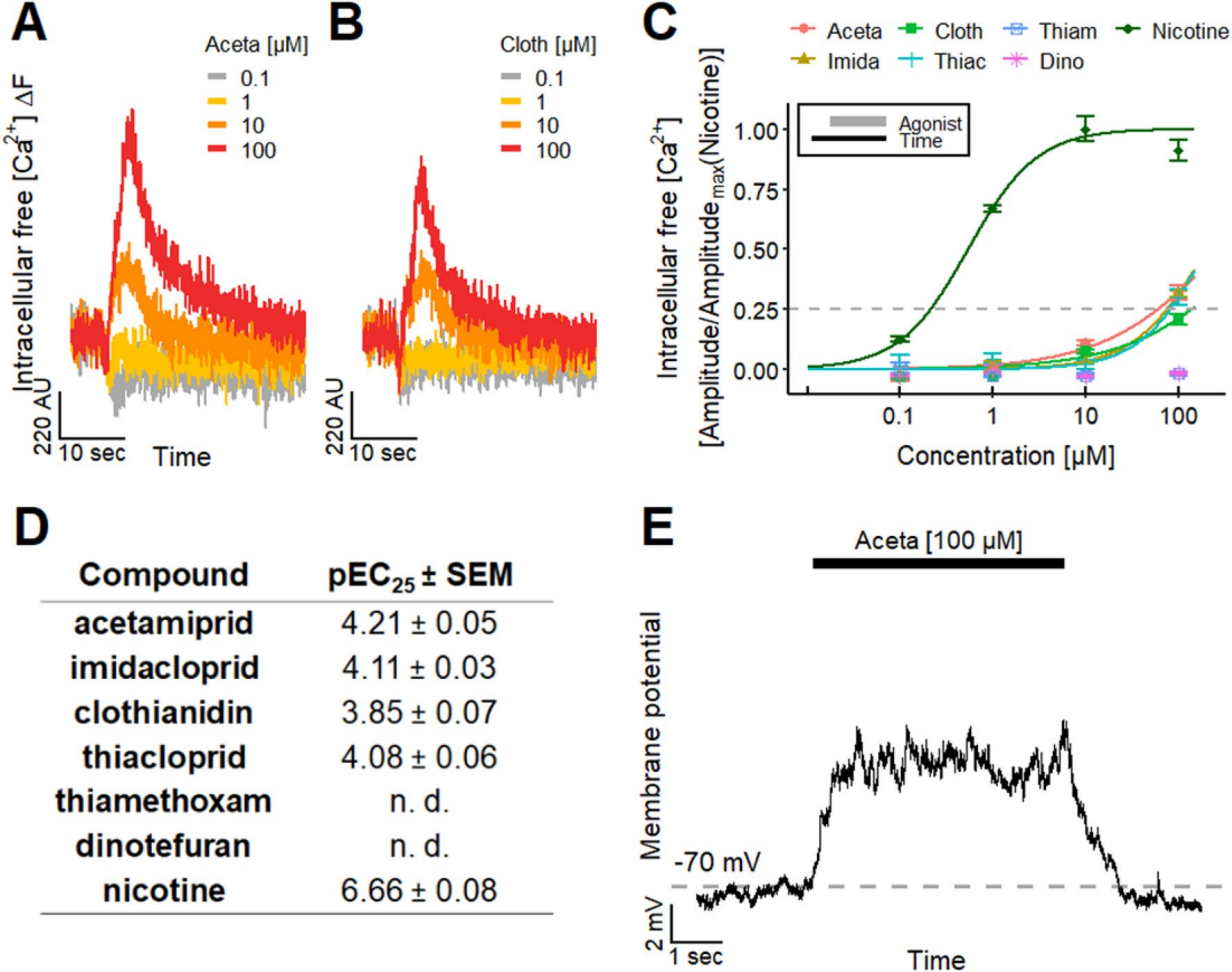
Effect of neonicotinoids on LUHMES neurons.** a**, **b** Traces of Ca^2+^-imaging show the effects of the neonicotinoids **a** Aceta and **b** Cloth on LUHMES neurons. **c** Concentration-dependent effect of the neonicotinoids Aceta, Imida, Cloth, Thiac, Thiam and Dino and the positive control nicotine. Amplitudes were normalized to the maximal amplitude evoked by nicotine. Note the treatment scheme (upper left corner), illustrating the experimental design. **d** Table with corresponding pEC_25_ values for the tested neonicotinoids and nicotine. Detailed data on n numbers are found in table S6. **e** Manual patch clamp recording of a long-lasting depolarization of the membrane potential during the application of 100 µM Aceta for 5 s (*n* = 4). The Aceta-induced depolarization from a holding potential of − 70 mV was not sufficient to evoke action potential firing

After demonstrating the presence of functional nAChRs on LUHMES and establishing the test system’s suitability to study nAChR-based toxicity, we investigated a subset of six neonicotinoids, namely acetamiprid (Aceta), imidacloprid (Imida), clothianidin (Cloth), thiacloprid (Thiac), thiamethoxam (Thiam) and dinotefuran (Dino), using Ca^2+^-imaging as endpoint (Fig. 4a, b). All compounds, except for Thiam and Dino, evoked responses, which we calibrated to the maximum effect observed at 10 µM nicotine (Fig. 4c). We determined pEC_25_ values, as the responses triggered by the neonicotinoids did not reach the 50% response level in the tested concentration range (≤ 100 µM). A comparison of the responses of the four active compounds to those of nicotine based on pEC_25_ values indicated that the pesticides had a two orders of magnitude lower potency, but triggered clear responses at 10–100 µM concentrations (Fig. 4d). Our data also indicate that the four active pesticides have lower potencies than nicotine or the endogenous neurotransmitter ACh. One straightforward explanation for the potency data observed may be the different affinities of the compounds for the set of nAChRs expressed on LUHMES cells. Binding assays using α4β2 nAChR have suggested such potency differences (Tomizawa and Casida 2005).

Our data show that also human cells react to neonicotinoids with changes of [Ca^2+^]_i_, as has been shown earlier for Aceta and Imida in other mammalian cells, i.e., primary rat neuronal cultures (Kimura-Kuroda et al. 2012). Effects of neonicotinoids on human neurons may have remained hitherto relatively unnoticed, as many tests only focus on endpoints related to cell viability and cell morphology. For instance, we did not observe any effect of neonicotinoids on the neurite outgrowth of LUHMES cells (Fig. S3). Our finding that human neuronal cells react to neonicotinoids demonstrates the importance of functional assays to assess potential adverse effects on neurons.

To confirm the important findings from Ca^2+^-imaging experiments, we additionally performed manual patch clamp recordings (Fig. 4e). Aceta (100 µM) clearly depolarized the cells, but not to a sufficient extent to trigger action potential firing (*n* = 4). The time course of depolarization gives some indication on the types of receptors involved: the long-lasting effect suggests that mainly non-α7 nAChRs contributed to the current (Mihalak et al. 2006; Rollema et al. 2007; Alijevic et al. 2020). With α7 nAChR activation as main mode of action, a rapid depolarization followed by a timely repolarization would have been observed during the application. However, it cannot be excluded from the data available that α7 nAChRs at least partially were co-activated, together with non-α7 nAChRs.

### ***Neonicotinoids effects on Ca***^***2***+^***-signaling of individual LUHMES neurons***

**Fig. 5:**
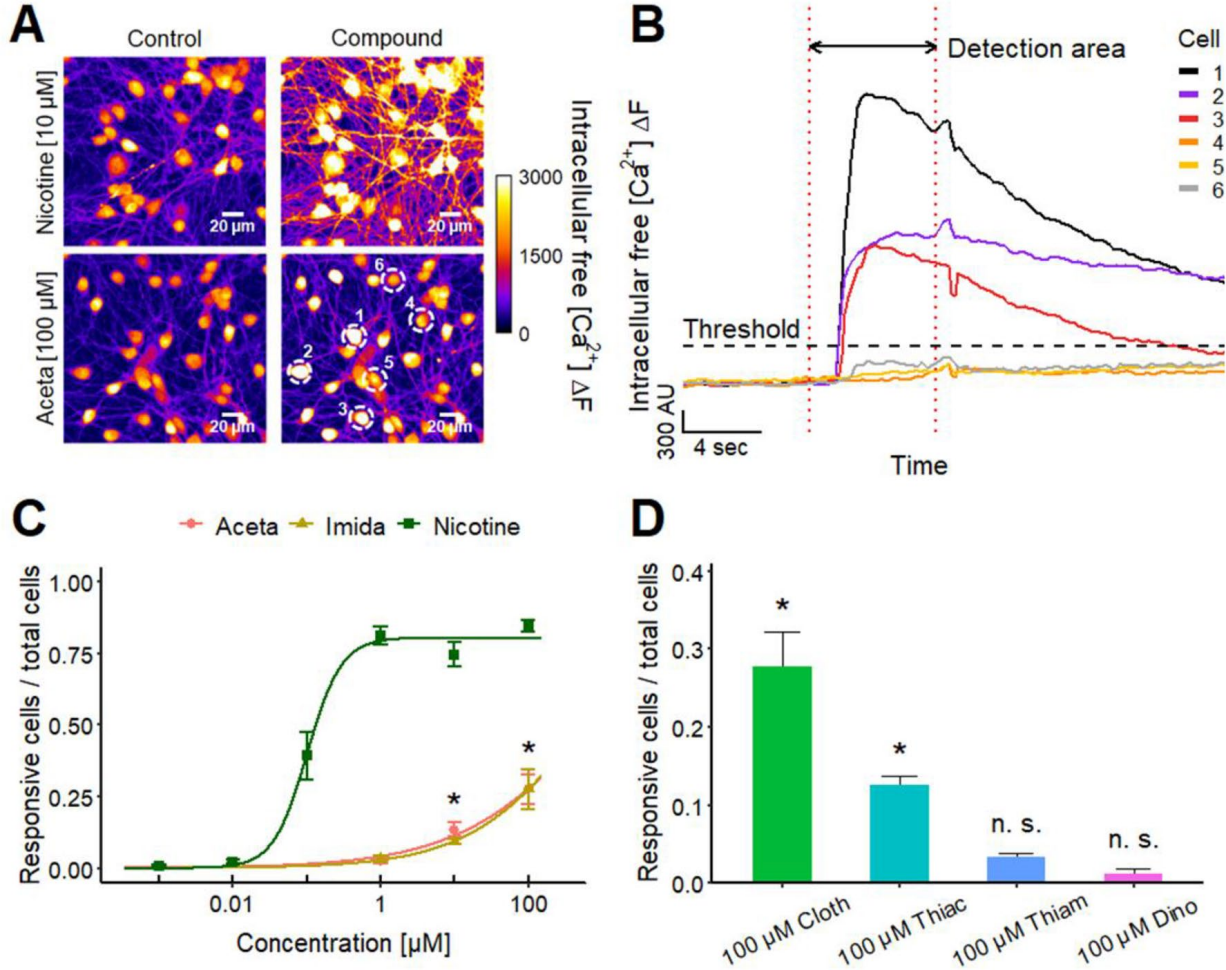
Effects of neonicotinoids on Ca^2+^-signaling on the level of individual neurons. **a** LUHMES neurons during control and during the application of 10 µM nicotine and 100 µM Aceta. Pictures of single-cell Ca^2+^-imaging recordings were taken with the Cell Observer (Carl Zeiss Microscopy). Images are shown in false color to enhance the interpretability. **b** Traces of single-cell Ca^2+^-imaging recordings of six cells (marked in **a**) with and without response to the application of 100 µM Aceta. Threshold for response detection is defined as mean + 3 × SD of negative control recordings. **c** Percentage of cells that responded to the application of nicotine, Aceta and Imida in single-cell Ca^2+^-imaging recordings. Changes are significant (*p < 0.05, *t* test) for 10 µM Aceta and Imida. Using more stringent ANOVA with Dunnett’s post hoc test, there was a significant difference for 100 µM, but only a trend (*p* > 0.05 for 10 µM). This range of effect significance agrees well with calculations of the Imida benchmark concentration (BMC_10_ = 11.2 µM) and its upper 95% confidence limit (BMCU_10_ = 26 µM). **d** Fraction of cells reacting to Cloth, Thiac, Thiam and Dino at a concentration of 100 µM. Note the enlarged *y*-axis. **c**, **d** Statistical significance was determined against negative control recordings (*, significant by ANOVA; n. s., not significant). Detailed data on n numbers and percentages of responsive cells are found in table S7 and S8, respectively

After our initial findings of neonicotinoid effects on human neuronal cultures (LUHMES), it was important to confirm [Ca^2+^]_i_ changes on the level of single cells. We used a Ca^2+^-imaging approach with high spatial resolution, and found that the positive control nicotine triggered a rapid rise in [Ca^2+^]_i_ in most neuronal cell bodies as well as in the neurite network (Fig. 5a). Aceta triggered a response only in a subset of cell bodies and in parts of the neurite network. Some cells clearly did not respond (Fig. 5a, b). The concentration-dependency of the responding cells was quantified for nicotine, Aceta and Imida (Fig. 5c). The curves looked similar to the concentration–response curves obtained with the high-throughput system (Fig. 4c). At 1 µM nicotine, the relative number of responsive cells reached its maximum of about 80%. For Aceta and Imida, cell responses were significantly increased at 10 µM, and they comprised about one-quarter of all cells at the highest test concentration of 100 µM. We also detected responses of the LUHMES neurons to the application of Cloth and Thiac, but not for Thiam and Dino at their highest tested concentration (Fig. 5d). To further confirm our findings from high-throughput imaging, we used Tubo also in single-cell recordings. The nAChR antagonist concentration-dependently reduced the number of cells responding to Aceta (100 µM, Fig. S4). In summary, these findings confirm the data obtained with high-throughput Ca^2+^-imaging measurements. They suggest an impact of Aceta, Imida, Cloth and Thiac on Ca^2+^-signaling of LUHMES neurons; they also confirm the low/absent effectiveness of Thiam and Dino. It remains unclear, why the otherwise quite homogeneous LUHMES cultures show response heterogeneity at the single cell level. Since LUHMES form an interactive network, different cells in the network differ by their interconnection, their neighborhood and their firing history, and all this may contribute to differential responsiveness to nicotine or neonicotinoids (Loser et al. 2021).

### The role of α7 nAChR for responses to neonicotinoids in LUHMES and SH-SY5Y neurons

**Fig. 6:**
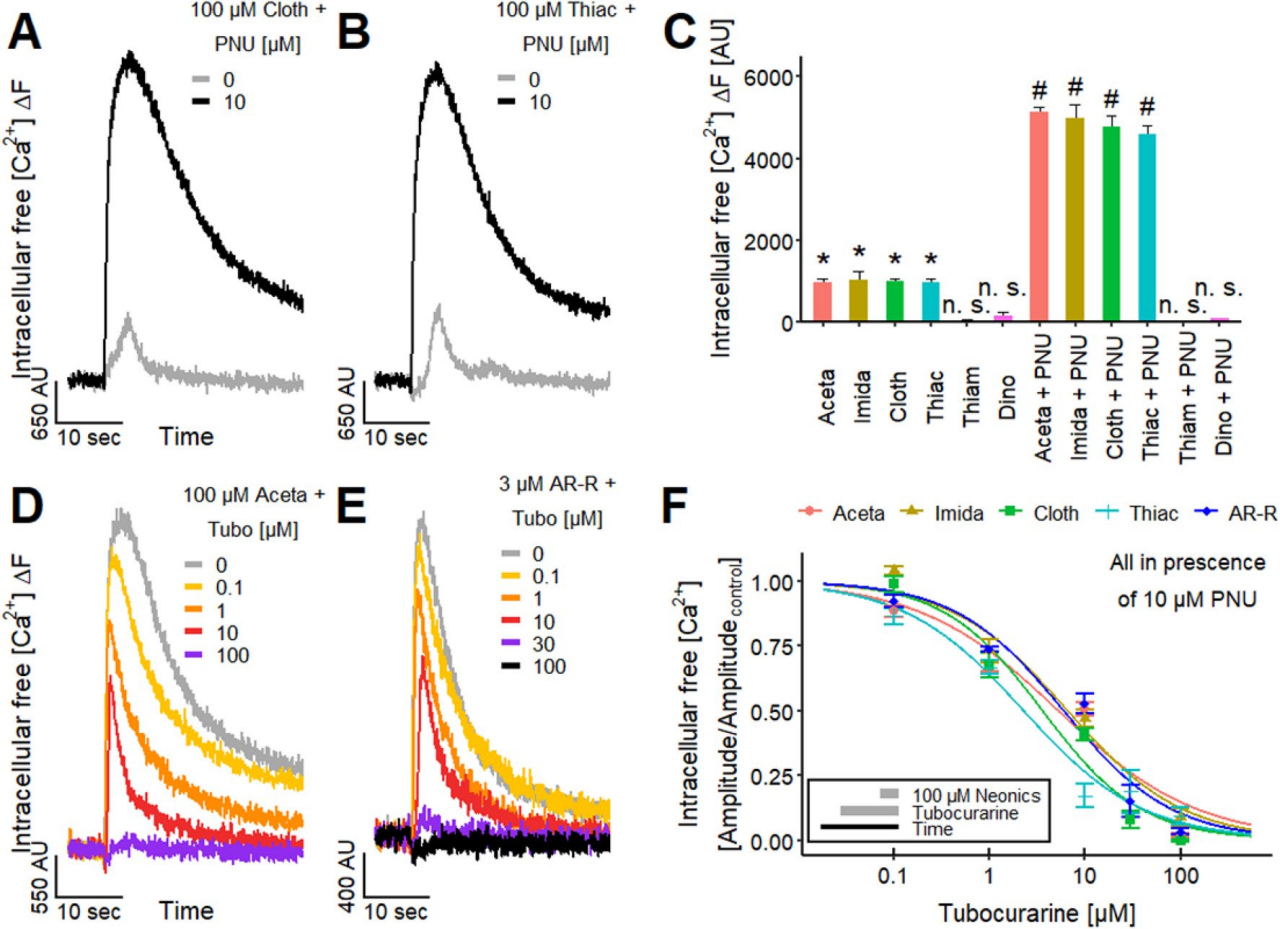
Effect of neonicotinoids on α7 nAChR.** a**, **b** Ca^2+^-imaging traces of the effects of the neonicotinoids **a** Cloth and **b** Thiac during control and in the presence of 10 µM PNU, which was preincubated for 4.5 min. **c** Effects of the neonicotinoids Aceta, Imida, Cloth, Thiac, Thiam and Dino (100 µM) on the Ca^2+^-imaging signal of LUHMES neurons in absence and presence of 10 µM PNU. Statistical significance was determined between the recordings of the neonicotinoids without PNU and negative control recordings (*, significant; n. s., not significant) and between the recordings of each neonicotinoid without and with PNU (#, significant; n. s., not significant).** d**, **e** Traces of Ca^2+^-imaging showing the effect of pre-applied tubocurarine (Tubo) on the signals evoked by **d** 100 µM Aceta and **e** 3 µM AR-R, both in the presence of pre-applied 10 µM PNU. **f** The concentration–response curves illustrate the effects of Tubo on the response of the LUHMES neurons to the acute exposure to the neonicotinoids Aceta, Imida, Cloth and Thiac (100 µM) and the α7 agonist AR-R (3 µM). All recordings were performed in the presence of 10 µM PNU, which was preincubated for 4.5 min. The resulting pIC_50_ values were 5.28 ± 0.10, 5.18 ± 0.09, 5.45 ± 0.08, 5.64 ± 0.08 and 5.22 ± 0.09 for Aceta, Imida, Cloth, Thiac and AR-R, respectively. Note the treatment scheme (lower left corner), illustrating the experimental design. Detailed data on n numbers are found in table S6

The α7 nAChR is widely distributed in the central nervous system and is involved in the modulation of neurotransmitter release (McGehee et al. 1995; Gray et al. 1996; Alkondon et al. 1999; Levin et al. 2006; Gotti et al. 2006; Zoli et al. 2015). To address the role of α7 nAChRs in neonicotinoid effects, we performed Ca^2+^-imaging experiments in the presence of PNU. While PNU had no effect by itself on non-stimulated cells, it drastically enhanced the [Ca^2+^]_i_-increase (peak and duration) triggered by, e.g., Cloth or Thiac (Fig. 6a, b). A systematic comparison of all six compounds used in our study showed that PNU significantly enhanced the responses to Aceta, Imida, Cloth and Thiac. Thiam and Dino had no effect, independent of the presence or absence of PNU (Fig. 6c). The enhancing effect of PNU indicates an activation of human α7 nAChRs by Aceta, Imida, Cloth and Thiac, but not Thiam and Dino. These data are in good agreement with a study by Cartereau et al. (2018). An agonist activity of Imida on α7 nAChRs has also been described in other systems (Yamamoto et al. 1998; Ihara et al. 2003).

**Fig. 7:**
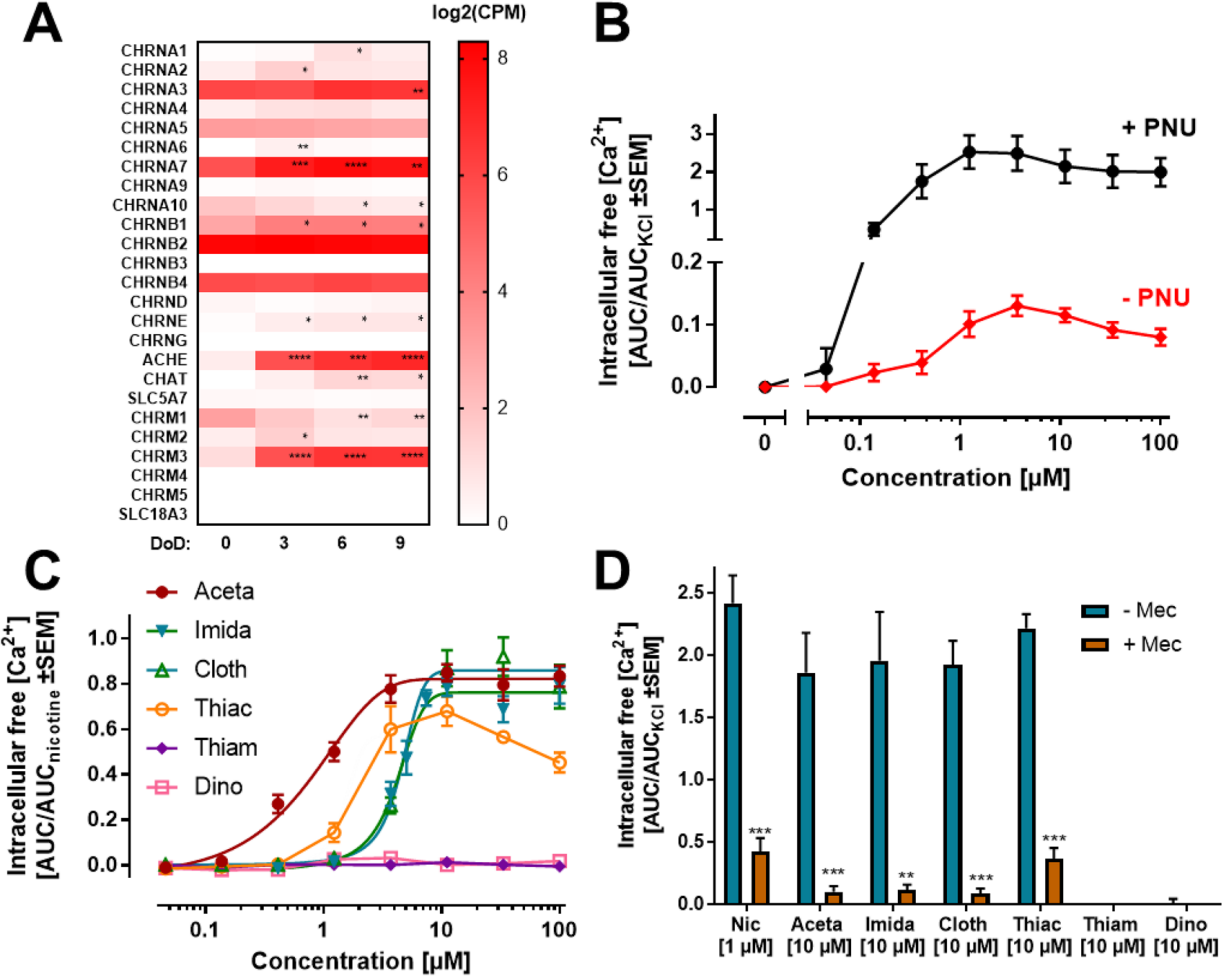
Nicotine signaling in SH-SY5Y cells. **a** The expression of genes coding for nAChR subunits was determined by whole-transcriptome RNA-sequencing during differentiation of SH-SY5Y cells. The raw counts were normalized to counts per million total counts (CPM) and log2-transformed. Significance of changes between day of differentiation (DoD) zero and DoD3-9 was evaluated by ANOVA with Dunnett’s multiple comparison test. *p < 0.05, **p < 0.01, ***p < 0.001, ****p < 0.0001. **b** The SH-SY5Y cells were differentiated for 72 h, and used on DoD3 for Ca^2+^-imaging experiments. The increase of the [Ca^2+^]_i_ triggered by nicotine was measured in the presence or absence of 10 µM PNU. The responses were evaluated as the area under the curve (AUC) of the increased fluorescence of the calcium-sensitive dye Fura-2 for 0–150 s after compound addition. An example of an original recording is shown in figure S5C. The responses were normalized to the [Ca^2+^]_i_ response of SH-SY5Y cells after depolarization with 30 mM KCl (AUC_KCl_) (*n* = 4–6). **c** The [Ca^2+^]_i_ response of SH-SY5Y cells triggered by Aceta, Imida, Cloth, Thiac, Thiam and Dino was measured in the presence of 10 µM PNU. The AUC of the response (0–150 s) was normalized to the AUC of the response evoked by the treatment of the cells with 11 µM nicotine (AUC_nicotine_) (*n* = 3). The estimated pEC_50_ values were 6.10 ± 0.07, 5.38 ± 0.03, 5.33 ± 0.01 and 5.73 ± 0.01 for Aceta, Imida, Cloth and Thaic, respectively. **d** The [Ca^2+^]_i_ responses of SH-SY5Y cells triggered by nicotine, Aceta, Imida, Cloth, Thiac, Thiam and Dino were measured in the presence of 10 µM PNU, and the presence or absence of Mec (125 µM). The AUC of the responses was normalized to the AUC_KCl_ (*n* = 3–5). Significance was evaluated by multiple *t* tests. *p < 0.05, **p < 0.01, ***p < 0.001

As the stimulation of human neuronal nAChRs has important toxicological implications, we checked main findings in a second, entirely independent test system: SH-SY5Y human neuroblastoma cells. Such cultures have been used earlier to prepare cell membranes containing nAChRs and also to measure the efflux of ^86^Rb^+^ (Lukas et al. 1993; Tomizawa and Casida 1999). Here, we characterized the receptor subunit expression profile during retinoic acid-induced differentiation, and we found particularly high and consistent (over time) levels of α7 (Figs. 7a and S5A). Upon exposure of 3-day differentiated SH-SY5Y cells to nicotine, a clear, but relatively low increase of [Ca^2+^]_i_ was observed, with a pEC_50_ value of about 7.0. This response was drastically augmented by the α7 stabilizer/allosteric modulator PNU. The increase in [Ca^2+^]_i_ triggered by nicotine in the presence of PNU was similar or even higher than the very strong response evoked by 30 mM KCl (Figs. 7b and S5C). In addition, the [Ca^2+^]_i_ responses stimulated by the neonicotinoids Aceta, Imida, Cloth and Thiac were significantly increased (in efficacy and in potency) in the presence of PNU (Fig. S5D). Under these conditions, the neonicotinoids Aceta, Imida, Cloth and Thiac triggered [Ca^2+^]_i_ responses with pEC_50_ values in the low µM range (Fig. 7c). Thiam and Dino had no effect, which fully confirmed our previous findings in LUHMES cultures (Fig. 6c). Cytotoxicity or major changes of receptor expression were not observed for nicotine, Aceta or Imida (Fig. S5A, B). In summary, the SH-SY5Y data provided clear evidence that a subclass of the tested neonicotinoids are agonists on the α7 nAChR, triggering a [Ca^2+^]_i_ response in human cells at low µM concentrations (Fig. 7c). The high potency of the neonicotinoids in the neuroblastoma test system might be explained by a high contribution of the α7 nAChR in these cells, and by the known left-shifting effect of the concentration–response curve by the positive allosteric modulator PNU. Such a potency shift was also observed in LUHMES stimulated with AR-R (Fig. 3c), and it is also known for other agonists (Hurst et al. 2005; Grønlien et al. 2007).

As specificity control for the above-described experiments, we checked whether the responses of nicotine and the four active neonicotinoids were blocked by Mec. The elimination of [Ca^2+^]_i_ responses under this condition confirms nAChRs as mediators (Fig. 7d). A similar control experiment was also performed in LUHMES cultures. The responses of all four active neonicotinoids, as well as AR-R, were blocked in the presence of PNU by nAChR antagonism (Fig. 6d–f). Thus, the data from two different experimental systems showed that a subgroup of neonicotinoids triggered Ca^2+^ signaling in human neurons.

### Molecular docking studies in support of observed functional effects

As there was a clear subgrouping of neonicotinoids concerning their functional effect on neurons, we explored whether structural commonalities/differences would support such activity differences. In a first step, some physicochemical parameters relevant to receptor interaction were compared. It was conspicuous that Dino is clearly the most hydrophilic compound of the set and that Thiam has a particularly high polar surface area (Fig. S6A). These extreme features may contribute to the fact that the two compounds behaved differently than the other four neonicotinoids, but the explanatory value of these data is quite limited.

Therefore, we took a more comprehensive approach to identify a possibly differential interaction of the compounds with the receptor. For this purpose, receptor models were established for the α7 nAChR and the α4ß2 nAChR, and molecular docking was performed for all six compounds. The docked poses for each compound were ranked and clustered according to their protein–ligand interaction fingerprints. Then, the highest populated clusters were analyzed (Fig. S6B). This revealed that Aceta, Imida, Cloth and Thiac align well in the known nicotine-binding site. The chloropyridine- and chlorothiazol-moieties of the pesticides pointed towards loops D and E from the complementary subunit and the electronegative nitro and cyano groups reaching out to the tip of the loop C (Fig. S6C). This binding behavior has also been described as “common binding mode”, and it has been described earlier (Tomizawa et al. 2008) for both Imida and nicotine, based on co-crystallization of these ligands with the homopentameric ACh binding protein complex (AChBP: the most established model for α7-related structures) (Ihara et al. 2008). Our docking study showed that Thiam almost exclusively exhibited an “inverted” binding mode with the electronegative nitroguanidine group placed close to the area where the other compounds position the chloroheteroaryl substructure, and vice versa (Fig. S6D). Dino showed a binding mode different from all the other compounds. This is most probably due to the lack of a chloroheteroaryl group (Fig. S6E). Thus, the molecular docking experiments indicate that the subgrouping might be determined by the predominant orientation of the compounds in the binding site. The set of high-quality (i.e., more likely) docking poses for Aceta, Imida, Cloth, and Thiac contained many solutions that correspond to the so-called common binding mode (similar to nicotine), but some also suggested an inverted binding mode. In the case of Thiam, only the inverted mode was observed. This might explain the low affinity of Thiam, as the inverted mode has been linked to lower binding affinity (Tomizawa et al. 2008). As Dino binding seemed to differ entirely from that of nicotine or the other compounds, a lowered affinity may be the consequence.

These observations give a molecular rationale for the observed functional differences. However, it is clear that they will require more detailed follow-up by dynamic docking models, including entropy considerations and binding energy calculations. Nevertheless, the preliminary findings presented here give already some potential explanations and better define future research needs.

### Modulation of cholinergic responses by neonicotinoids

**Fig. 8:**
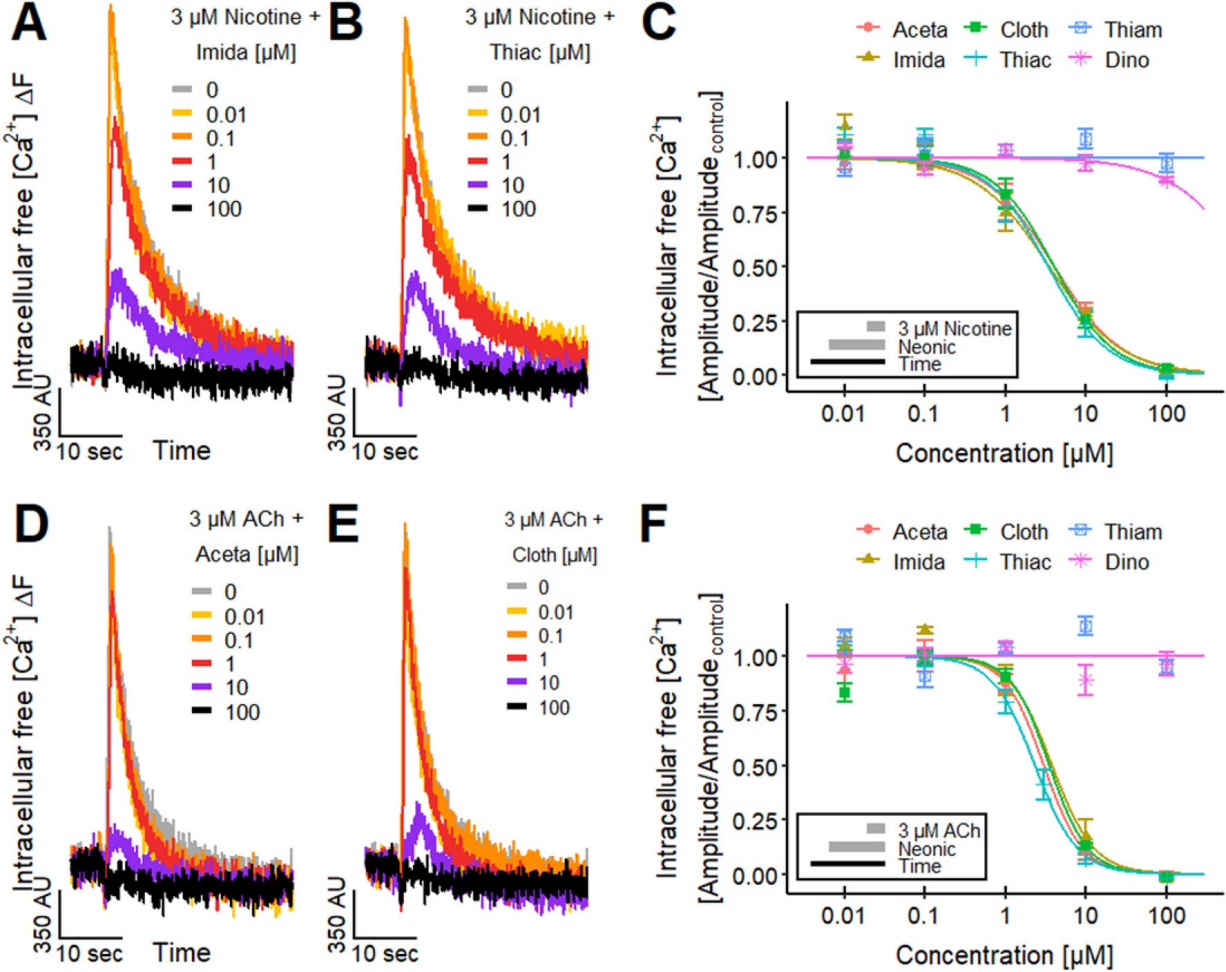
Effect of neonicotinoids on responses evoked by nicotine and ACh.** a**, **b** Ca^2+^-imaging traces displaying the responses of LUHMES neurons to the acute exposure to 3 µM nicotine in the presence of the neonicotinoids **a** Imida and **b** Thiac, which were preincubated for 4.5 min. **c** Concentration-dependent effects of the pre-applied neonicotinoids Aceta, Imida, Cloth, Thiac, Thiam and Dino on the nicotine-evoked responses. The resulting pIC_50_ values were 5.40 ± 0.08, 5.47 ± 0.10, 5.41 ± 0.07 and 5.48 ± 0.08 for Aceta, Imida, Cloth and Thiac, respectively. Thiam and Dino did not show an effect.** d**,** e** Ca^2+^-imaging traces showing the effects of the neonicotinoids **d** Imida and **e** Thiac, which were preincubated for 4.5 min, on the signals evoked by the application of 3 µM ACh. **f** Effects of the pre-applied neonicotinoids Aceta, Imida, Cloth, Thiac, Thiam and Dino on the response of LUHMES neurons triggered by 3 µM ACh. The pIC_50_ values were 5.53 ± 0.09, 5.43 ± 0.08, 5.46 ± 0.07 and 5.64 ± 0.04 for Aceta, Imida, Cloth and Thiac, respectively. Thiam and Dino had no effect. Note the treatment schemes (lower left corner), illustrating the experimental design. Detailed data on n numbers are found in table S6

An important feature of nAChR signaling is tachyphylaxis (self-inactivation of the receptor during the signaling process). Under such conditions, signaling may stop even in the presence of a ligand, and signaling cannot be repeated within a certain period after an initial stimulation. This complex behavior is also termed desensitization (Fenster et al. 1997; Quick and Lester 2002; Paradiso and Steinbach 2003; Lester 2004; Rollema et al. 2010; Marks et al. 2010; Capelli et al. 2011; Papke et al. 2011; Campling et al. 2013; Eaton et al. 2014; Arias et al. 2015; Rollema and Hurst 2018). We investigated tachyphylaxis to obtain further evidence for the action of neonicotinoids via the nAChRs. In our experimental setup, the neonicotinoids were added at various concentrations to LUHMES cultures, and thereafter, the [Ca^2+^]_i_ responses triggered by nicotine were recorded. In cells pretreated with the neonicotinoids, a strong attenuation of the nicotine signaling was observed (Fig. 8a, b). A quantification of the concentration-dependency of the down-modulation resulted in pIC_50_ values of ~ 5.4 for Aceta, Imida, Cloth and Thiac (Figs. 8c, S7A). Thiam and Dino did not show a negative modulation on the nicotine-induced response of LUHMES neurons, again indicating/confirming that they do not interact with nAChRs. Experiments performed in SH-SY5Y cells confirmed these results (Fig. S5E).

Another set of desensitization experiments was performed using the endogenous neurotransmitter ACh as stimulus. The resulting pIC_50_ values for response attenuation were ~ 5.5 for Aceta, Imida, Cloth and Thiac. Thiam and Dino had no effect (Fig. 8d–f). For Imida, a similar desensitizing effect on ACh-evoked responses has been reported for insect nAChRs (Oliveira et al. 2011) earlier, and such tachyphylaxis phenomena are wide-spread and well documented for ionotropic cholinergic receptors in general (Fenster et al. 1997; Quick and Lester 2002; Paradiso and Steinbach 2003; Lester 2004; Rollema et al. 2010; Marks et al. 2010; Capelli et al. 2011; Papke et al. 2011; Campling et al. 2013; Eaton et al. 2014; Arias et al. 2015; Rollema and Hurst 2018). The different effects of the neonicotinoids Aceta, Imida, Cloth and Thiac in comparison with Thiam and Dino on the nicotine- and ACh-triggered responses are fully consistent with the differences in agonist activity.

We also explored the issue of a potential cross-tolerance with other receptor systems. This might occur by, e.g., affecting intracellular processes related to Ca^2+^-signaling. We tested the impact of neonicotinoid pretreatment on signaling via the ionotropic purinergic receptor subtype P2X3, which has been shown to be functionally expressed in LUHMES (Loser et al. 2021). The response evoked by the agonist α,β-meATP (Bianchi et al. 1999; Khakh and North 2012; Loser et al. 2021) was not affected by the pre-applied neonicotinoids or nicotine (Fig. S7B). This suggests that the tolerance mediated by the neonicotinoids for nicotine is triggered directly on the level of nAChRs.

The desensitizing activity of an agonist occurs typically at concentrations that can activate the receptor, as tachyphylaxis is a typical consequence of receptor activation. However, it is also possible that low concentrations, not sufficient to trigger significant receptor activation may desensitize a receptor. In the latter case, a ligand can have a higher potency for desensitization than for activation of the nAChR (Fenster et al. 1997; Paradiso and Steinbach 2003; Lester 2004; Rollema et al. 2010; Capelli et al. 2011; Arias et al. 2015; Rollema and Hurst 2018). Here, we observed indeed a left-shift of potency in the concentration–response relationship. We determined the lowest active concentration, assuming a benchmark response of 10% (BMC_10_). These BMC_10_ values were obtained from the concentration–response curves using a log-logistic model. They ranged between 0.37 and 0.62 µM for the four active compounds. The upper end of their 95% confidence interval (the BMCU_10_), i.e., concentrations having a high likelihood to trigger a biological effect, were at 0.87–3.6 µM (Table S9). These experiments confirm that effects of Imida and related compounds on human neuronal nAChRs are likely to occur in the low µM range.

For confirmation of the desensitizing effect of the neonicotinoids on nAChRs on single cells, we performed Ca^2+^-imaging experiments on a microscope stage. High or low concentrations of Aceta (10 µM or 100 µM) were applied prior to a nicotine (10 µM) stimulus. The voltage-gated Na^+^ channel modulator veratridine (VTD) was used as a positive control at the end of the stimulation series, to verify unaltered overall cell excitability and to exclude possible unspecific effects of Aceta on the electrical activity of the cells. Aceta (100 µM) significantly reduced the percentage of responding cells to the ensuing addition of nicotine, whereas the response to VTD was not affected (Fig. S8). These observations support our previous results from high-throughput imaging (Figs. 8C, S7A).

### Tolerance triggered by neonicotinoids against the selective agonist ABT 594

**Fig. 9:**
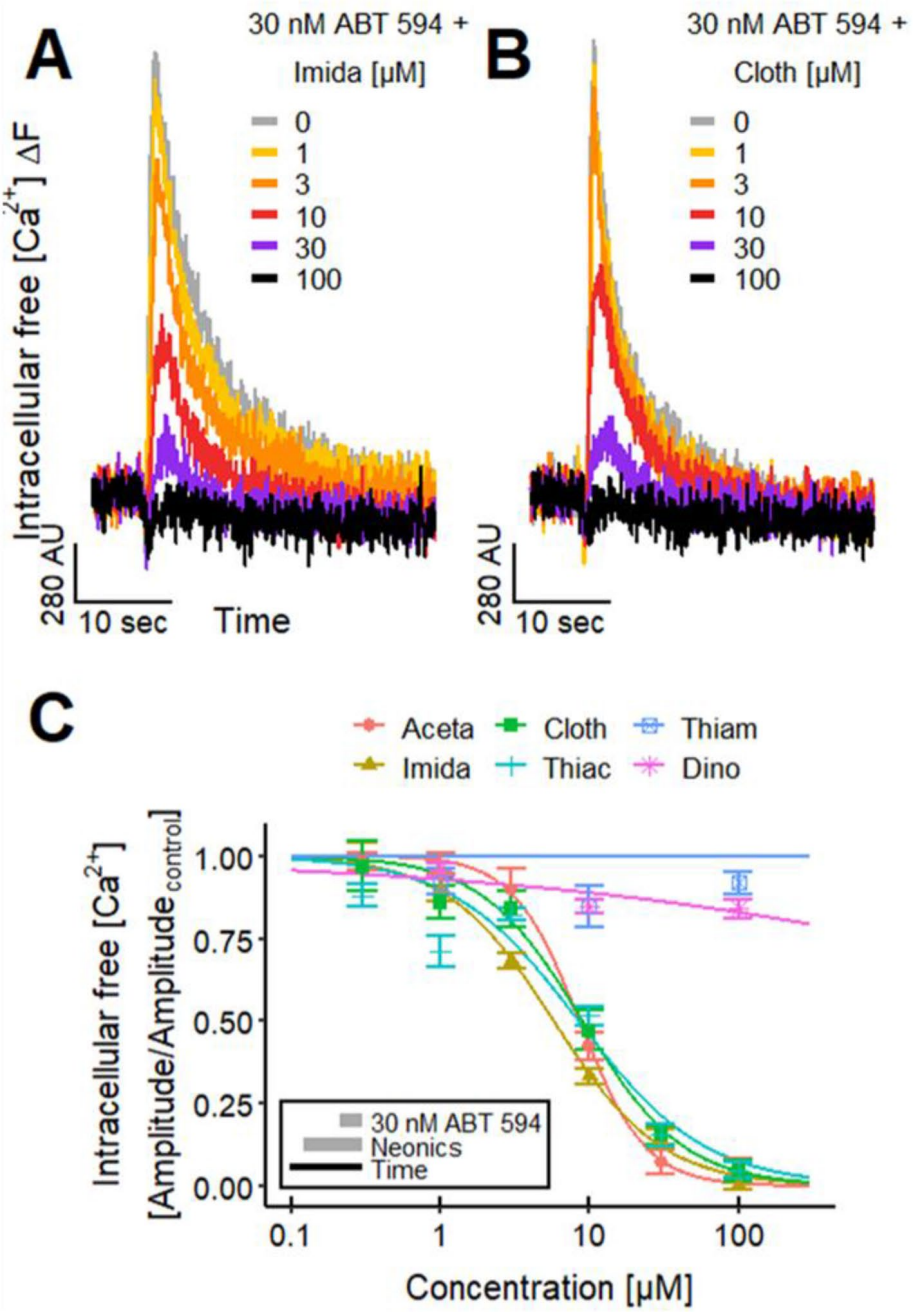
Effect of neonicotinoids on responses evoked by ABT 594.** a**,** b** Ca^2+^-imaging traces of the effects of the neonicotinoids **a** Imida and **b** Cloth, which were preincubated for 4.5 min, on the responses stimulated by the acute exposure to 30 nM ABT 594. **c** The concentration-dependent effects of the pre-applied neonicotinoids Aceta, Imida, Cloth, Thiac, Thiam and Dino on the ABT 594-induced response resulted in pIC_50_ values of 5.07 ± 0.03, 5.24 ± 0.03, 5.05 ± 0.05 and 5.08 ± 0.06 for Aceta, Imida, Cloth and Thiac, respectively. No pIC_50_ values could be determined for Thiam and Dino. Note the treatment scheme (lower left corner), illustrating the experimental design. Detailed data on n numbers are found in table S6

In a final set of experiments, we asked whether the desensitizing effect of the neonicotinoids necessarily involved α7 nAChR. Therefore, cell pretreated with the neonicotinoids were exposed to the non-α7 nAChR agonist ABT 594. The pIC_50_ values for response attenuation were ~ 5.1 for Aceta, Imida, Cloth and Thiac (Fig. 9a–c). When given at a concentration of 100 µM, the compounds blocked the response induced by 30 nM ABT 594 completely. As seen in the previous experiments, Thiam and Dino did not show any modulatory effect. These results further confirm our previous findings, as the pIC_50_ values were in a similar range as the values obtain for stimulations with nicotine and ACh (Fig. 8).

### Exposure considerations and in vitro-to-in vivo comparisons

When exposure to neonicotinoids is considered, at least three scenarios need to be distinguished (Cimino et al. 2017): intentional exposure, occupational (non-intentional) exposure, normal dietary exposure. Several studies demonstrate a wide-spread use of the compounds for suicidal attempts, and under such conditions, several grams of active pesticide are ingested (Mohamed et al. 2009). While occupational exposure is usually low under most conditions in Europe due to restricted use (closed processes for seed treatment implemented), there are reports on hundreds of symptomatic cases (Marfo et al. 2015). For the general population, acute reference doses (ARfD) have been set by EFSA to, e.g., 25 µg/kg/day for Aceta and 80 µg/kg/day for Imida. Monitoring studies (e.g., in 2018 in Europe (European Food Safety Authority (EFSA) et al. 2020)) found for Aceta a maximal acute exposure of 57 µg/kg/day. Thus, assuming that it is realistic that the ARfD can be exceeded twice, a realistic maximal exposure on a single day may be 114 µg/kg (Aceta) to 160 µg/kg (Imida). In rodent studies, the NOAELs for chronic endpoints was 7–15 mg/kg for Aceta and this point-of-departure was used also for setting the ARfD due to uncertainties/limitations related to the regulatory developmental neurotoxicity study (EFSA Panel on Plant Protection Products and their Residues (PPR) 2013).

The internal exposure data are relatively limited. For mice, treated with NOAEL levels of Imida and Aceta (10 mg/kg), the brain levels were around 3–6 ppm, and plasma concentrations were in a similar range (15–30 µM) (Ford and Casida 2006). Human data are mostly available for urinary metabolites used for biomonitoring, and the concentrations were maximally in the low nM range (Zhang et al. 2019; Li et al. 2020; Li and Kannan 2020). Due to the lack of more direct data, we built physiology-based toxicokinetic (PBTK) models to predict plasma and brain concentrations (Table S11). An exposure to Imida (0.16 mg/kg; corresponding to a maximally expected level in the normal population; see above) was predicted to lead to plasma concentrations in the 0.8–1.6 µM range and to brain concentrations of 0.5–1.2 µM (Fig. S9). Reverse modelling showed that a brain concentration of 2 µM Imida (considered a point-of-departure from our in vitro studies) would be reached after an intake of 0.2 mg/kg body weight in the average population. The in vitro–in vivo comparisons yield plausible data, in the sense that normal population exposure would normally yield sub-µM concentrations, and that such concentrations would not be sufficient to trigger nAChRs. Vice versa, an uptake exceeding the ARfD is predicted to lead to concentrations that may be sufficient to affect nAChR signaling.

## Conclusions and outlook

In the present study, we investigated the functional expression and impact of nAChRs on [Ca^2+^]_i_ in two well-established model systems for dopaminergic human neurons. Based on this characterization, we asked whether the most widely used neonicotinoids (Jeschke et al. 2011; Bass et al. 2015) affected human nAChRs, in addition to their known action on insect receptors (Brown et al. 2006; Tan et al. 2007). We provided here compelling evidence for the triggering of cholinergic signal transduction by the neonicotinoid pesticides Aceta, Imida, Cloth and Thiac. Thus, our study is in line with earlier findings obtained with other mammalian cells, i.e., primary rat brain cultures (Kimura-Kuroda et al. 2012), and it provides clear evidence for a potential human hazard of such compounds.

Many subtypes of nAChRs are expressed in dopaminergic neurons, where they modulate the electrical activity and the release of dopamine (Rapier et al. 1988; Grady et al. 1992; Quik and Kulak 2002; Mameli-Engvall et al. 2006; Quik and Wonnacott 2011; de Kloet et al. 2015). As the nicotinic signaling thereby affects the functioning, plasticity and development of the dopaminergic nervous system, it is of crucial importance to learn about a potential effect of neonicotinoids in neurons and to determine the risk potential for the dopaminergic system or other important circuits of the brain (Stevens et al. 2003; Wheeler and Cooper 2004; Welsby et al. 2006; Slotkin et al. 2006; Ziviani et al. 2011; Miwa et al. 2011; Lozada et al. 2012; Romoli et al. 2019). While our study provides unambiguous evidence for the agonist role of some neonicotinoids on human nAChRs, follow-up studies will be required to judge the full toxicological implication of our findings. We provide here some initial conclusions and highlight important gaps of knowledge to be addressed.

Concerning the toxicant–target interaction (also termed molecular initiating event, using the terminology of adverse outcome pathways), two findings are remarkable. First, it is clear from a broad range of data provided, that various subtypes of nAChR may be activated by neonicotinoids. We provide here evidence for a role of both α7 and non-α7 receptors. This is consistent with binding experiments and functional studies based on ^86^Rb^+^-flux, all of which demonstrated the interaction of, e.g., Imida or Aceta with various receptor types (Tomizawa and Casida 1999, 2005). Notably, the available literature data on the potencies of Imida and Aceta have a wide spread (0.7–320 µM, depending on the assessment method). Our data on a physiological signaling response (change of [Ca^2+^]_i_) provide important information on toxicologically important concentration ranges. Our data on direct stimulation of naive cells by neonicotinoids suggest that the lowest concentrations eliciting a neuronal response are in the 10 µM range. However, it needs to be considered that [Ca^2+^]_i_ signaling effects in our model systems may be masked by extremely rapid receptor desensitization. Prevention of tachyphylaxis, combined with allosteric enhancement by the modulator PNU, allowed Aceta, Imida, Cloth and Thiac to be detected in SH-SY5Y cells at concentrations of ≥ 1 µM. This potency range is fully consistent with data from a desensitization assay in LUHMES cells, where the functional consequence of neonicotinoid exposure on subsequent cholinergic signaling was investigated. In addition, in this setup, compound activity started to be detected in the very low micromolar range for all four active compounds. Notably, the desensitizing aspect may be equally problematic for neurodevelopment as a potential overstimulation. To use such data for in vitro-to-in vivo extrapolations and as point-of-departure for risk assessment, the corresponding free toxicant concentrations need to be known: biokinetics calculations applied to our cell models showed that the free drug concentration was very close (mostly < 1% deviation) to the nominal concentration (Table S10).

The second important aspect of target interaction is our observation of two distinct subgroups of neonicotinoids with respect to neuronal signaling. While four test compounds showed relatively similar effects and effect potencies, two other agents (Thiam, Dino) were inactive within the test range (i.e., at least 100 × less potent). The activity differences correlated well with favored docking poses of the ligands in a molecular model of the nAChR. While this will need further elucidation on the level of receptor binding and other molecular pharmacology approaches, this clear subgrouping suggests a high specificity of the LUHMES assay.

Whether the signaling disturbances measured here have lasting neurofunctional effects needs to be studied further. However, many studies on other nicotinic agonists (including nicotine) suggest that compounds triggering nAChR will affect the nervous system function not only acutely, but also affect its plasticity and development (Wheeler and Cooper 2004; Slotkin et al. 2006; Ziviani et al. 2011; Lozada et al. 2012; Romoli et al. 2019). One study in rat neurons also shows that Aceta/Imida may alter the gene expression of neurons upon prolonged exposure (Kimura-Kuroda et al. 2016). Concerning human data, four studies on chronic effects of neonicotinoids have been reported (Cimino et al. 2017). In all of them, some significant general developmental/neurological effects were observed when the population was stratified according to urine biomarkers, proximity to agricultural production or to use of neonicotinoid-containing anti-tick sprays.

A more difficult question is whether neonicotinoids have the potential to affect the development of the fetal brain (EFSA Panel on Plant Protection Products and their Residues (PPR) 2013) and thus trigger developmental neurotoxicity (DNT). Many other compounds that trigger alterations in neurotransmitter signaling, but no overt structural defects have been shown to trigger DNT. They include for instance MDMA, heroine (Aschner et al. 2017) and nicotine itself (Slikker Jr et al. 2005; Dwyer et al. 2009; Slotkin et al. 2016). Moreover, compounds that hardly affect the adult brain (in typical exposure situations) have been shown to affect the developing brain with late life consequences of fetal exposure. Well-documented examples are methylmercury and lead (Grandjean and Landrigan 2014). These examples from other compounds make it plausible that neonicotinoids pose a DNT hazard, but direct evidence is quite limited at present. As is good practice in toxicology, each individual compound needs to be evaluated for its proper effect/hazard. A transfer of knowledge and conclusions from one compound (e.g., nicotine) to others (neonicotinoids) always bears uncertainties (Rovida et al. 2020). More definite data on individual neonicotinoids are required in the future to confirm or disprove the DNT alert triggered by our study.

For a full risk assessment, some additional aspects need to be addressed. First, the molecular structure–activity relationship will require new technical approaches. Receptors with defined subunit stoichiometry may be expressed in *Xenopus laevis* oocytes to clearly identify the molecular targets of active neonicotinoids and to verify that the inactive ones do not interact with any of the receptors. Second, metabolism and metabolites will require additional attention. In general, neonicotinoids have relatively long half-lives, and we have provided data on the direct activity of parent compounds. However, there may be also some active metabolites. Several studies showed that the Imida metabolite desnitro-imidacloprid exhibits higher potency at inhibiting the binding of [^3^H]nicotine or [^125^I]α-BGT to mammalian nAChRs compared to its precursor Imida, but similar to nicotine (Chao and Casida 1997; Tomizawa and Casida 1999; D’Amour and Casida 1999; Tomizawa et al. 2000). The same finding has also been reported for chicken α4β2 nAChR expressed in M10 cells (Tomizawa and Casida 2000). Thus, generation of toxicokinetics data on this metabolite together with bioactivity measurements on human neurons will be interesting. Third, PBTK models for all compounds will need to be applied to different exposure scenarios. A model validation with human data would be desirable to show reliability of the data and predictions from these models. Previous attempts into this direction have used microdosing in volunteers with deuterium-labeled compounds (Harada et al. 2016). Concerning the question of neurological consequences, controlled short-term experiments with volunteers have been performed to study effects of nicotine (Grundey et al. 2018). Whether such studies would be ethically acceptable for pesticides is doubtful for most countries in Europe.

## Supplementary Information

Below is the link to the electronic supplementary material.

Supplementary file1 (DOCX 1838 kb)

## Abbreviations

ACh: Acetylcholine
Aceta: Acetamiprid
ARfD: Acute reference dose
AR-R: AR-R 17779
AUC: Area under the curve
BMC: Benchmark concentration
[Ca^2^^+^]_i_: Intracellular free calcium concentration
cAMP: N6,2′-0-dibutyryl 3′,5′-cyclic adenosine monophosphate
Cloth: Clothianidin
Dino: Dinotefuran
DMSO: Dimethyl sulfoxide
Imida: Imidacloprid
Mec: Mecamylamine
MLA: Methyllycaconitine
nAChR: Nicotinic acetylcholine receptor
pEC_25_: Negative logarithm of the quarter maximal effective concentration
pEC_50_: Negative logarithm of the half-maximal effective concentration
pIC_50_: Negative logarithm of the half-maximal inhibitory concentration
PBTK: Physiology-based toxicokinetic
PLO: Poly-l-ornithine
PNU: PNU-120596
Thiac: Thiacloprid
Thiam: Thiamethoxam
Tubo: Tubocurarine
VTD: Veratridine
