# Supplementary material for: Functional alterations by a subgroup of neonicotinoid pesticides in human dopaminergic neurons
Source: Arch Toxicol. 2021 Mar 29;95(6):2081–107. doi: 10.1007/s00204-021-03031-1 (PMC8166715; doi:10.1007/s00204-021-03031-1)
Supplement: Supplementary file 1 — Supplementary file1 (DOCX 1838 kb) [file 204_2021_3031_MOESM1_ESM.docx]

**Supplementary information for**

**Functional alterations by a subgroup of neonicotinoid pesticides in human dopaminergic neurons**

*Dominik Loser^1,2,3^, Maria G. Hinojosa^5^, Jonathan Blum^3^, Jasmin Schaefer^1,2^, Markus Brüll^3^, Ylva Johansson^5^, Ilinca Suciu^3^, Karin Grillberger^6^, Timm Danker^1,2^, Clemens Möller^4^, Iain Gardner^7^, Gerhard F. Ecker^6^, Susanne H. Bennekou^8^, Anna Forsby^5^, Udo Kraushaar^1,#^, Marcel Leist^3,#^*

^1^ NMI Natural and Medical Sciences Institute at the University of Tübingen, 72770 Reutlingen, Germany

^2^ NMI TT GmbH, 72770 Reutlingen, Germany

^3^ In vitro Toxicology and Biomedicine, Dept. inaugurated by the Doerenkamp-Zbinden foundation, University of Konstanz, 78457 Konstanz, Germany

^4^ Life Sciences Faculty, Albstadt-Sigmaringen University, 72488 Sigmaringen, Germany

^5^ Department of Biochemistry and Biophysics, Stockholm University, 106 91 Stockholm, Sweden

^6^ University of Vienna, Department of Pharmaceutical Sciences, Vienna, Austria

^7^ CERTARA UK Limited, Simcyp Division, Level 2-Acero, 1 Concourse Way, Sheffield, S1 2BJ, United Kingdom

^8^ Technical University of Denmark, Kongens Lyngby, Denmark

^#^ These authors contributed equally

| **Table of Contents** | | |
| --- | --- | --- |
| **Fig. S1** | Gene expression profile of LUHMES cells for cholinergic components. | page 3 |
| **Fig. S2** | Effects of PNU-120596 on LUHMES neurons. | page 4 |
| **Fig. S3** | Impact of neonicotinoids on neurite outgrowth of LUHMES neurons. | page 5 |
| **Fig. S4** | Inhibition of acetamiprid (Aceta) triggered responses of LUHMES neurons by tubocurarine. | page 6 |
| **Fig. S5** | Effects of neonicotinoids on SH-SY5Y cells. | page 7-8 |
| **Fig. S6** | Physicochemical properties and molecular docking studies. | page 9-10 |
| **Fig. S7** | Effects of neonicotinoids on responses of LUHMES to selective agonists. | page 11 |
| **Fig. S8** | Effects of acetamiprid (Aceta) on Ca^2+^-signaling of individual LUHMES neurons. | page 12 |
| **Fig. S9** | Physiologically-based toxicokinetic modelling of imidacloprid concentrations in the human population. | page 13 |
| **Table S1** | Compound list. | page 14 |
| **Table S2** | Overview of pEC_50_ values for agonist experiments. | page 15 |
| **Table S3** | Overview of pIC_50_ values for antagonist experiments. | page 16 |
| **Table S4** | Overview of concentrations and replicates for agonist and antagonist experiments. | page 17 |
| **Table S5** | Overview of pIC_50_ values for experiments with neonicotinoids. | page 18-19 |
| **Table S6** | Overview of concentrations and replicates for experiments with neonicotinoids. | page 20-22 |
| **Table S7** | Overview of concentrations and replicates for single-cell Ca^2+^-imaging. | page 23 |
| **Table S8** | Overview of percentages of responsive cells of single-cell Ca^2+^-imaging. | page 24 |
| **Table S9** | Benchmark responses (BMR) for the neonicotinoids desensitization of nicotine and acetylcholine signaling. | page 25 |
| **Table S10** | Prediction of free drug concentrations in recording buffer of LUHMES. | page 26 |
| **Table S11** | Input parameters used in the Imidacloprid PBTK model. | page 26-27 |
|  | References | page 28 |


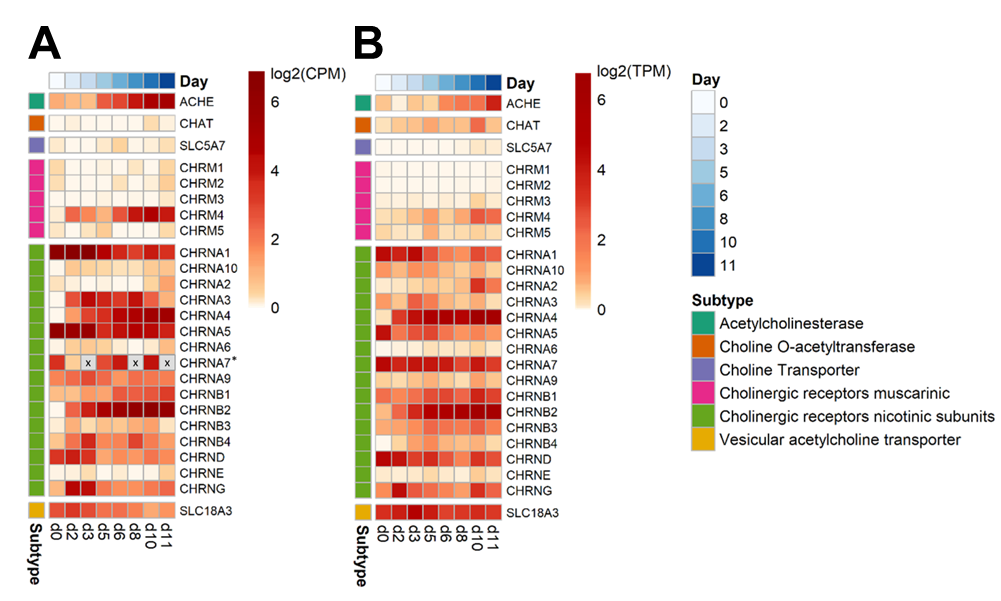


**Fig. S1: Gene expression profile of LUHMES cells for cholinergic components.**

Five biological replicates were generated from LUHMES cells differentiated for 2, 3, 5, 6, 8, 10, and 11 days, as well as from undifferentiated LUHMES cells (day 0). The heatmaps visualize the normalized and logarithmic counts for each gene (rows) of cholinergic components, and day of differentiation (columns, indicated in blues). Darker red indicates high expression, orange indicates low expression, and white indicates no expression. The genes are clustered by the gene group (e.g. receptor subtype). Gene groups are indicated by color in the first column. Samples were analyzed via (A) the TempO-Seq assay and via (B) traditional whole genome RNA-sequencing (RNAseq). The TempO-Seq analysis uses a part of the CHRNA7 mRNA sequence complementary to bases 374-424 (corresponding to amino acids 125-141). As data with this probe were unclear, we used qPCR as verification method (indicated by an asterisk). For this particular data set, the color code indicates the expression of the CHRNA7 receptor subunit gene relative to GAPDH. The red intensity was set in a way that an expression level corresponding to 10% of the GAPDH expression level (i.e. a very high level) was assigned the same hue as a log2(CPM) of 6  for the TempO-Seq method. Fields marked with “x”: no data was obtained for these days.


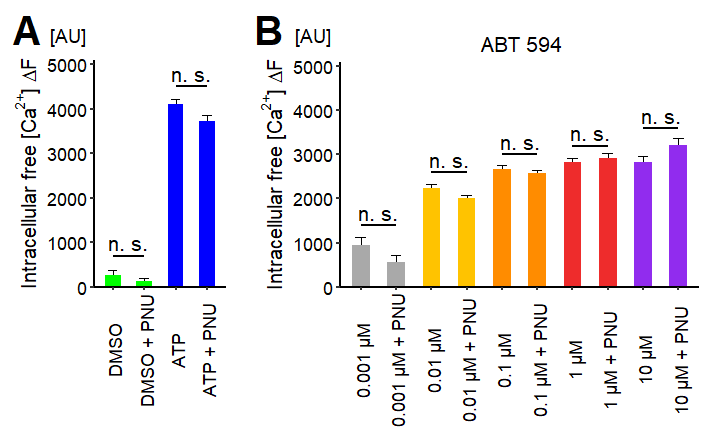


**Fig. S2: Effects of PNU-120596 on LUHMES neurons.**

(A-B) Ca^2+^-imaging experiments to examine the effects of PNU-120596 (PNU), a selective positive allosteric modulator of α7 nAChR, on LUHMES neurons. The cells were pretreated with 10 µM PNU for 4.5 min. (A) Effect of the absence and presence of 10 µM PNU on the responses induced by the application of 0.1% DMSO (DMSO, n ≥ 5), as vehicle control, and 1 µM α,β-meATP (ATP, n = 6), a selective P2X receptor agonist. (B) Influence of the absence and presence of 10 µM PNU on the responses evoked by the addition of ABT 594 (n = 6), a neuronal non-α7 nAChR agonist. Statistical significance was determined between recordings without and with PNU present (n. s., not significant).


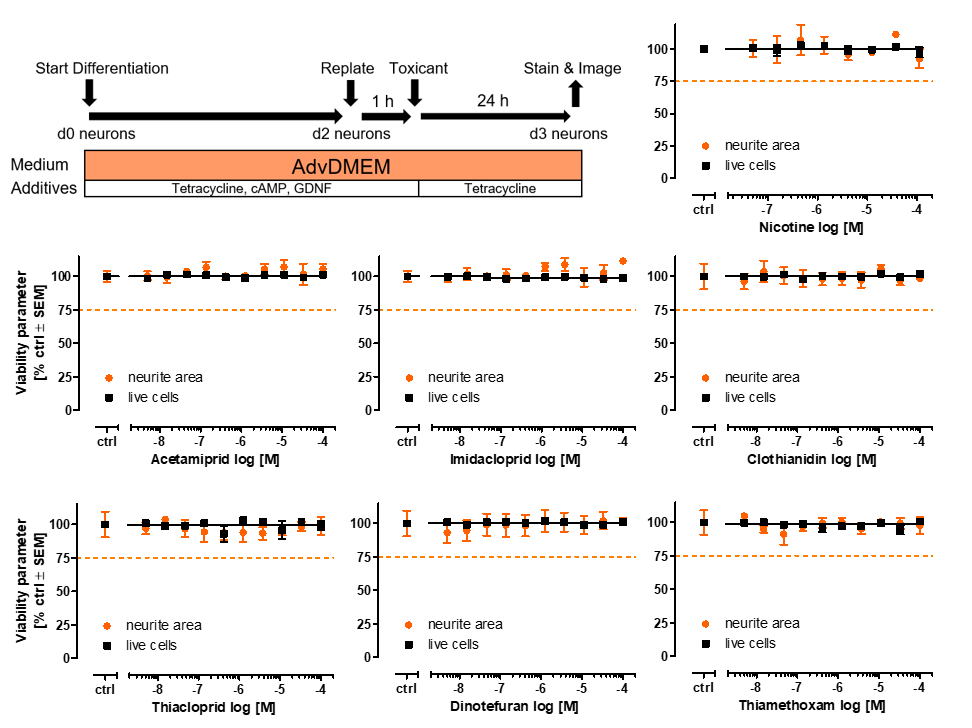


**Fig. S3: Impact of neonicotinoids on neurite outgrowth of LUHMES neurons.**

LUHMES cells were differentiated for two days. On day 2 of differentiation (d2 neurons) cells were replated and after 1 h of attachment, cells were treated for 24 h with nicotine and different neonicotinoids (5 nM - 100 µM). High content imaging was performed and live cells (black) and neurite area (orange) were assessed in parallel. All data are means ± SEM from at least two biological replicates. Dashed line indicates 75% of control.

**
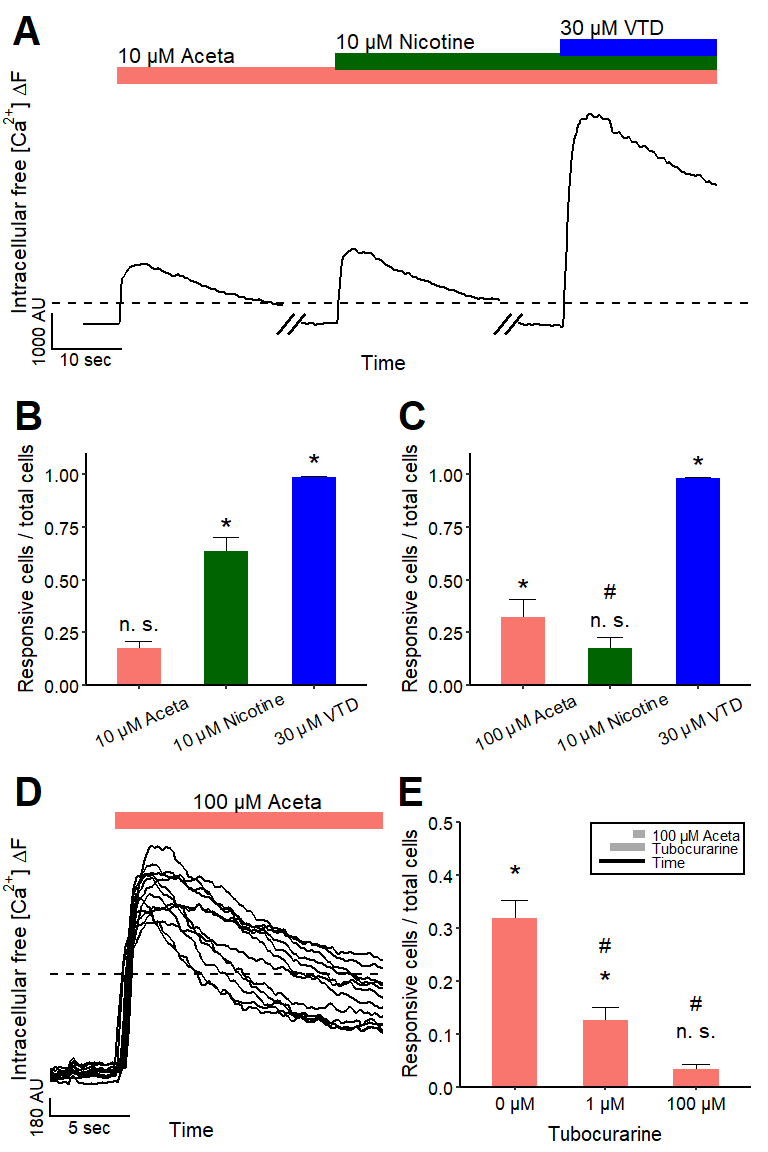
**

**Fig. S4: Inhibition of acetamiprid (Aceta) triggered responses of LUHMES neurons by tubocurarine.**

Percentage of cells that responded in single-cell Ca^2+^-imaging to the application of 100 µM Aceta in the absence and presence of the nAChR antagonist tubocurarine. Statistical significance was determined against negative control recordings (*, significant; n. s., not significant) and against the control recordings with 100 µM Aceta in the absence of tubocurarine (0 µM) (#, significant). Note the treatment scheme (upper right corner), illustrating the experimental design and the enlarged y-axis. Detailed data on n numbers and percentages of responsive cells are found in table S7 & S8, respectively.

**
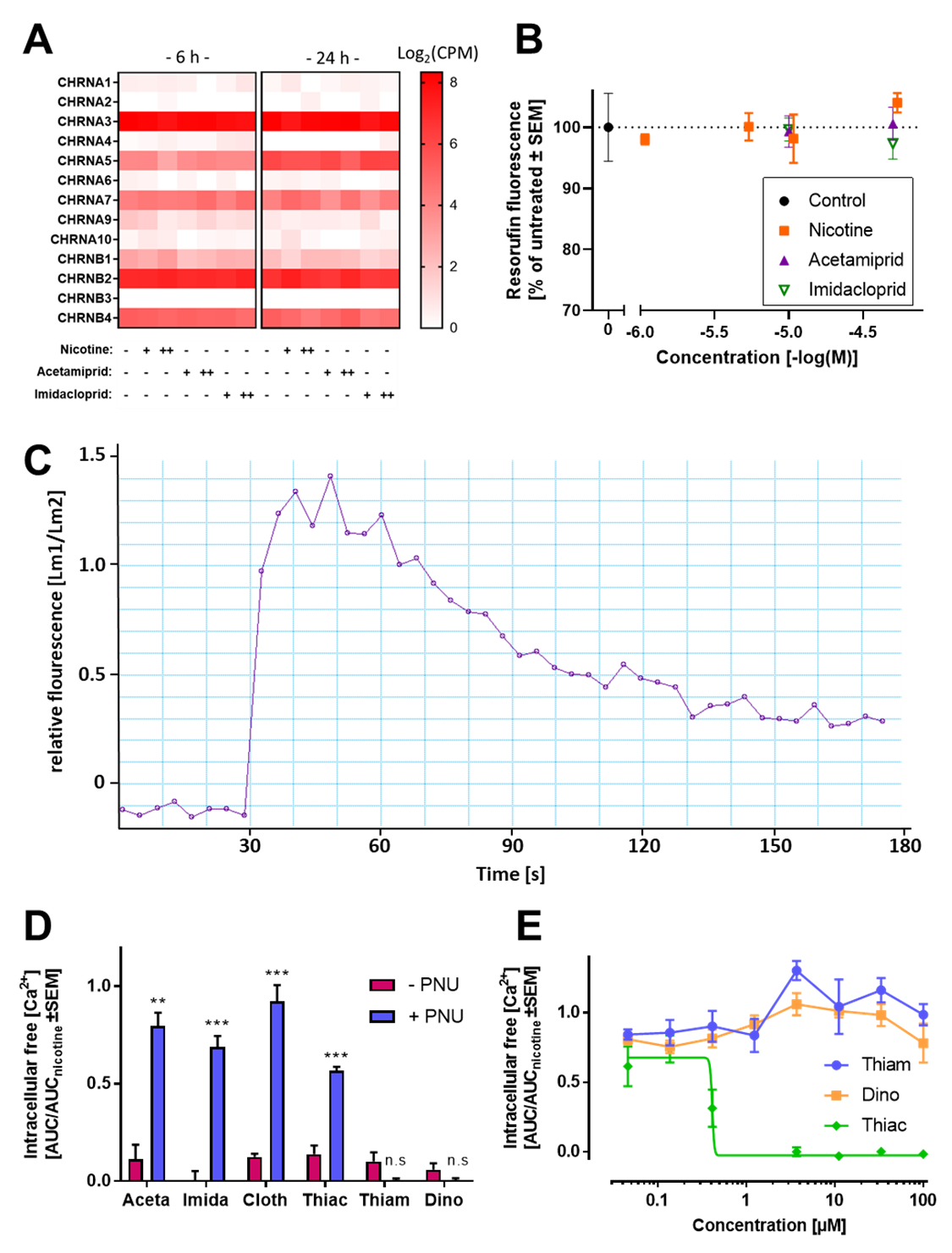
**

**Fig. S5: Effects of neonicotinoids on SH-SY5Y cells.**

(A) SH-SY5Y cells were treated with nicotine, acetamiprid, or imidacloprid on DoD3. After 6 and 24 h, samples for TempO-Seq analysis were taken. Counts were normalized to total counts per million and log2 transformed. – ≙ not present, Nicotine: + ≙ 1µM ++ ≙ 5 µM, acetamiprid/imidacloprid: + ≙ 10 µM ++ ≙ 50 µM. Data represent mean of ≥4 replicates from 2 experimental occasions. No significant differences between treatment and control were observed when p<0.05. (B) Cell viability of SH-SY5Y cells was determined by a resazurin assay. Cells were treated on DoD3 for 24 h. Resorufin fluorescence was normalized to untreated controls. (C) Example curve of the increased Fura-2 fluorescence after [Ca^2+^]_i_ response of SH-SY5Y triggered by 30 mM KCl. Cells were treated on DoD3. (D) Effects of neonicotinoids (33.3 µM) in absence and presence of 10 µM PNU. The responses to Aceta, Imida, Cloth and Thiac were strongly increased. Significance was evaluated by multiple t-tests. *p<0.05, **p<0.01, ***p<0.001. (E) The [Ca^2+^]_i_ responses triggered by the acute exposure to nicotine (11.1 µM) was negatively modulated by pre-applied Thiac. The pre-exposure to Thiam and Dino did not modulate the nicotine-evoked response.


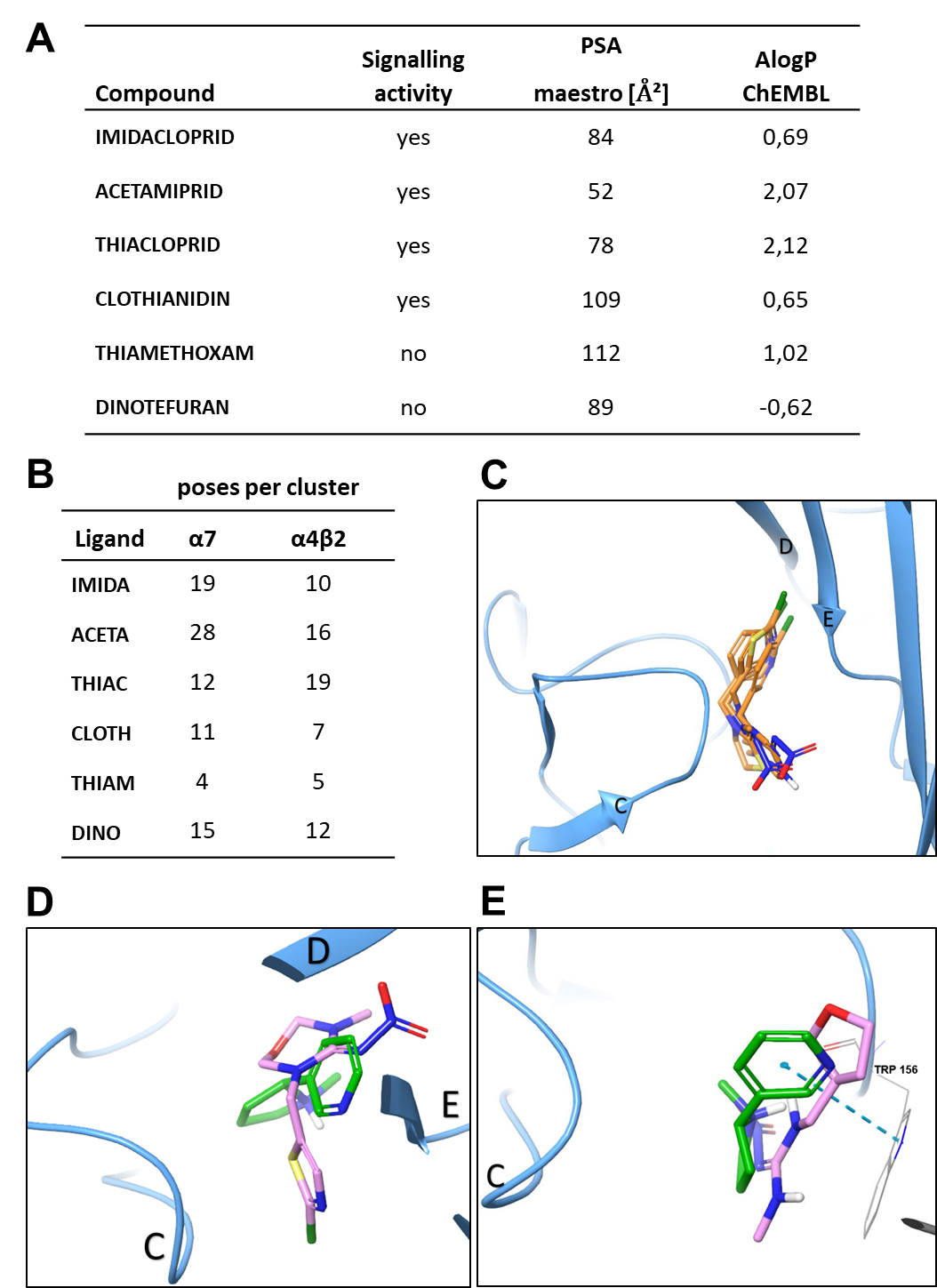


**Fig. S6: Physicochemical properties and molecular docking studies.**

(**A**) The octanol:water distribution coefficient (given as AlogP) and polar surface area (PSA) were calculated as mentioned in the methods section. In contrast to the other compounds, dinotefuran reported a negative AlogP value (i.e. higher hydrophilicity than the other compounds). The structurally similar compounds thiamethoxam and clothianidin had the highest values for PSA. (**B**) Up to 80 docked poses per ligand and per nAChR isoform were generated during the induced fit docking procedure. The poses were subsequently clustered according to the interaction-fingerprint of the docking-output. For each isoform, the highly populated clusters were analyzed further, and exemplary docking poses are displayed in C-E. (**C**) The neonicotinoids were docked to the α7 nAChR and to the α4β2 receptor at the α4β2 interface and at the α4α4-interface. The representative poses of imidacloprid, thiacloprid, acetamiprid and clothianidin in the binding site of the α4α4-interface are superimposed. The poses looked essentially similar in the other modelled receptor binding sites; for better view only one ribbon from the docked complexes is shown in blue. For spatial orientation in Figures C-E: the loops (and associated beta-sheet regions) A-C belong to the principal (+) binding subunit (e.g. alpha-4), while structures labelled D-F belong to the complementary (-) subunit (e.g. beta-2). Ligands: Chlorine-atoms are depicted in green, nitrogen-atoms in blue, sulfur-atoms in yellow, carbon-atoms in orange and nitrogen-atoms in blue. Chloroheteroaryl-moieties of the ligands are aligning well and are pointing towards loop D and E from the complementary subunit while the electronegative nitro- or cyano-functionalities are pointing towards the tip of the loop C of the principal chain. This binding orientation is outlining the “common” binding mode, which is also reported in co-crystallized homologous ACh-binding protein (AChBP)-complexes. (**D**) Thiamethoxam (pink structure) exhibits an “inverted” binding mode with the nitroguanidine-group being placed reversed with respect to the heteroarylic substructure, which is pointing towards the tip of loop C in this binding conformation. Nicotine (green structure) has been docked as reference compound into the homo-pentameric α7-structure and shows a “common” nicotinoid binding mode, with the amino-feature pointing away from loop C. (**E**) The α4(+)-β2(-)-interface is shown: Dinotefuran (pink structure) accommodates a distinct binding mode compared to the other compounds. The reference compound nicotine (green structure) is shown as it was actually found in the co-crystallized complex. In this common binding mode it undergoes stabilizing pi-stacking-interactions with a Tryptophan (Trp156) of the B-loop of the binding site. Such an interaction was found here to be impossible for dinotefuran (lack of the heteroarylic moiety; altered orientation).


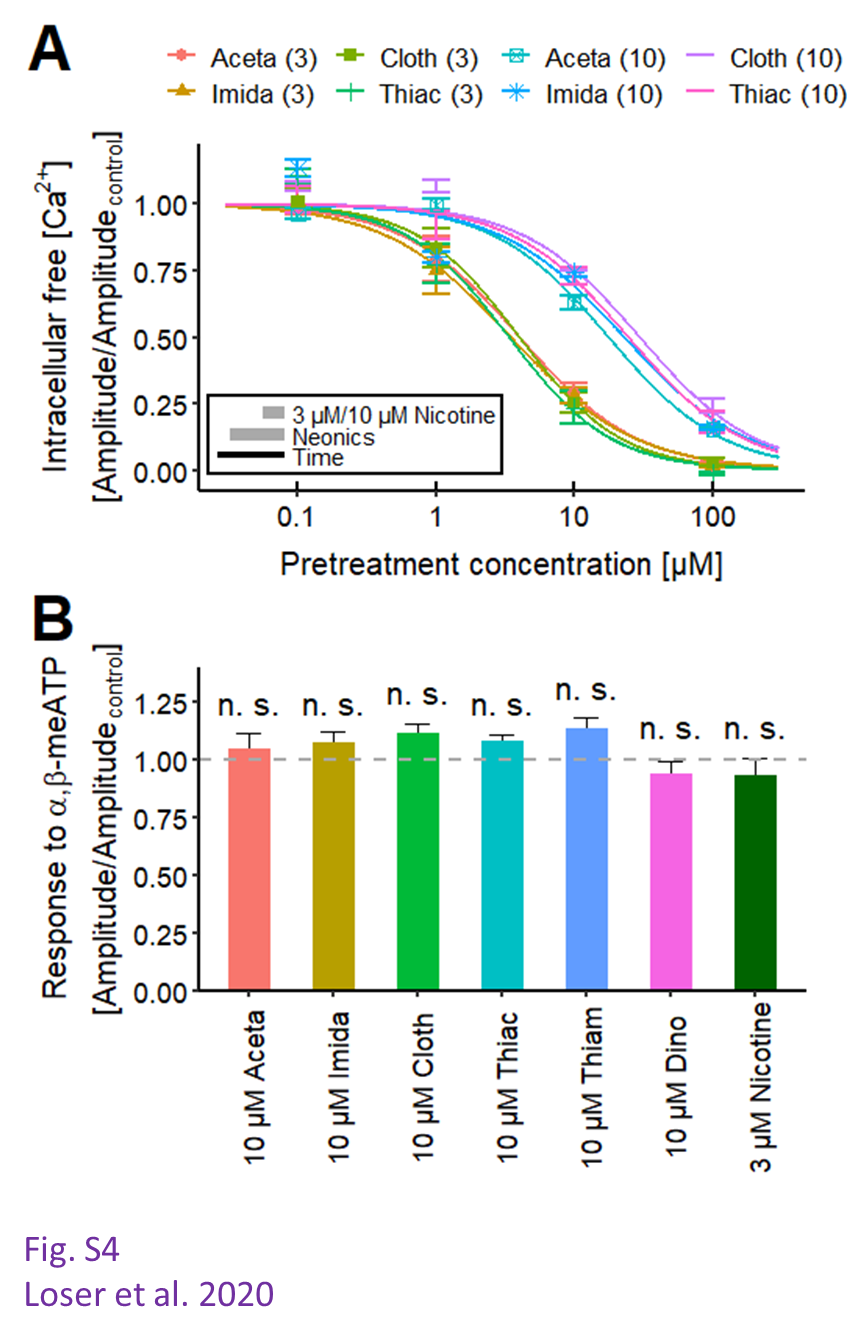


**Fig. S7: Effects of neonicotinoids on responses of LUHMES to selective agonists.**

(A) The concentration-response curves present the effects of the neonicotinoids Aceta, Imida, Cloth and Thiac, which were preincubated for 4.5 min, on the response of LUHMES neurons to the addition of 3 µM (3) and 10 µM (10) nicotine. The resulting pIC_50_ values are ~5.4 for the effects of Aceta, Imida, Thiac and Cloth on the response evoked by 3 µM nicotine, as described in fig. 8C. The pIC_50_s for the experiment with 10 µM nicotine are ~4.6 for Aceta, Imida, Cloth and Thiac. Detailed data on pIC_50_ values and n numbers are found in table S5 & S6, respectively. The impact of the four neonicotinoids was significantly different between the recordings with 3 µM and 10 µM nicotine. Note the treatment scheme (lower left corner), illustrating the experimental design. (B) Investigation of unspecific effects of neonicotinoids and nicotine on the responses evoked by 0.1 µM α,β-meATP, a P2X receptor agonist, using Ca^2+^-imaging. The neonicotinoids Aceta (n = 5), Imida (n = 5), Cloth (n = 5), Thiac (n = 5), Thiam (n = 6) and Dino (n = 6) and nicotine (n = 7) were preincubated for 4.5 min prior to the application of α,β-meATP. Statistical significance was determined against control recordings (n = 6; n. s., not significant).


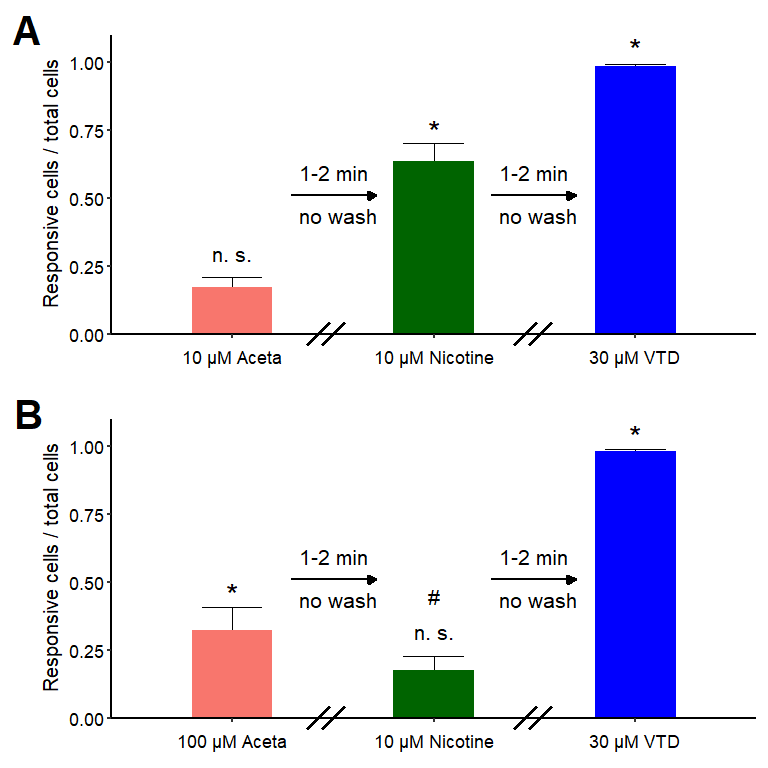


**Fig. S8: Effects of acetamiprid (Aceta) on Ca^2+^-signaling of individual LUHMES neurons.**

Percentage of responsive cells during the serial application of (A) 10 µM or (B) 100 µM Aceta, followed by the addition of 10 µM nicotine and 30 µM veratridine (VTD) in single-cell Ca^2+^-imaging. Compounds were incubated for 1-2 min, before the next application. The Na_V_ channel modulator VTD was used as a positive control. Statistical significance was determined against negative control recordings (*, significant; n. s., not significant) and between the values of 10 µM nicotine (#, significant). Detailed data on n numbers and percentages of responsive cells are found in table S7 & S8, respectively.


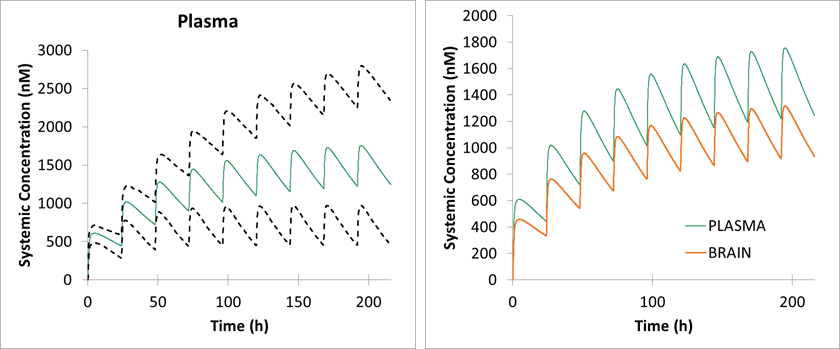


**Fig. S9: Physiologically-based toxicokinetic modelling of imidacloprid concentrations in the human population.**

A physiology-based toxicokinetic model (PBTK model) was established in the Simcyp Simulator V19 (Certara, Sheffield, UK), using a previously published approach (Albrecht et al. 2019). The input parameters for imidacloprid are given in table S11. The systemic exposure following an oral dose of 0.16 mg/kg, given every 24 h intervals to 100 individuals (50% female) with randomly assigned phenotypic and genotypic properties typical for a Caucasian Northern European population are shown. In the panel to the left, the predicted mean plasma concentrations (green line) are shown with the 5^th^ and 95^th^ percentiles of the population represented as dashed lines. In the panel to the right, mean plasma and brain concentrations are compared. A description of the full model building and model application for all compounds will be reported elsewhere, as this would exceed the scope of the current study.

**Table S1. Compound list.**

**Table S2. Overview of pEC_50_ values for agonist experiments.**

**Table S3. Overview of pIC_50_ values for antagonist experiments.**

**Table S4. Overview of concentrations and technical replicates for agonist and antagonist experiments.**

**Table S5. Overview of pIC_50_ values for experiments with neonicotinoids.**

(Table continued on next page.)

**Table S5. Overview of pIC_50_ values for experiments with neonicotinoids. (continued)**

**Table S6. Overview of concentrations and technical replicates for experiments with neonicotinoids.**

(Table continued on next page.)

**Table S6. Overview of concentrations and technical replicates for experiments with neonicotinoids. (continued)**

(Table continued on next page.)

**Table S6. Overview of concentrations and technical replicates for experiments with neonicotinoids. (continued)**

**Table S7. Overview of concentrations and technical replicates for single-cell Ca^2+^-imaging.**

**Table S8. Overview of percentages of responsive cells of single-cell Ca^2+^-imaging.**

**Table S9. Benchmark responses (BMR) for the neonicotinoids desensitization of nicotine and acetylcholine signaling.**

Overview of the BMR10 and BMR25 concentrations (in log[M]) of the Ca^2+^ response induced by nicotine and acetylcholine after an initial stimulation of LUHMES cells with neonicotinoids. For each BMR, the benchmark concentrations (BMC) and their confidence interval (BMCL and BMCU) are shown; n.a.: BMR could not be determined due to low/no effect within the tested concentration range.

**Table S10. Prediction of free drug concentrations in recording buffer of LUHMES.**

The free drug concentrations [µM] were calculated for the exemplary nominal concentrations of 1, 10 and 50 µM, based on the known recording buffer content of lipids and proteins and the number of cells used in each well. Calculations were performed as detailed in Kisitu et al. (2020) and Fisher et al. (2019).

**Table S11. Input parameters used in the imidacloprid PBTK model.**

| **Parameter** | **Value** | **Method/Reference** |
| --- | --- | --- |
| Molecular weight (g/mol) | 255.7 | Pubchem |
| log P | 0.4 | Predicted *in silico^b^.* |
| Compound type | Neutral |  |
| blood/plasma ratio [B/P] | 0.954 | measured |
| free fraction [fu] | 0.724 | measured |
| Main plasma binding protein | Human serum albumin | assumed |
| **Absorption parameters:^b^** |  |  |
| fa | 0.95 |  |
| (Table continued on next page.) | |  |
| **Table S11. Input parameters used in the Imidacloprid PBTK model. (continued)** | | |
| ka (1/h) | 1.16 |  |
| fu_gut_ | 1 | assumed |
| Q_gut_ (L/h) | 18.6 |  |
| P_eff,man_ (10^-4^cm/s) | 2.82 |  |
| P_trans,0_ (10^-6^cm/s) | 35.4 |  |
| **Distribution Model** | Minimal PBTK Model |  |
| V_SS_ (L/kg) | 0.85 | Predicted ^c^ |
| Brain: Plasma ratio | 0.75^d^ |  |
| Kp Scalar | 2 | Optimized |
| Enzyme | CYP3A4 |  |
| CL_int_ (μL/min/pmol) | 0.003^e^ |  |
| CL_R_ (L/h) | 0.17^f^ |  |

^a^: Predicted according to a mechanistic permeability model (Sugano 2009)

^b^: Average of 5 estimates from different source models

^c^: Method 2 (Rodgers and Rowland 2007)

^d^: Assumed same as mouse (Ford and Casida 2006)

^e^: Retrograde calculation based on predicted Vss and half-life of renal elimination reported for deuterated imidacloprid in humans (Harada et al. 2016)

^f^: Estimated from total CL and renal excretion of deuterated imidacloprid in human urine as reported by Harada et al. (2016).

**References:**

Albrecht W, Kappenberg F, Brecklinghaus T, et al (2019) Prediction of human drug-induced liver injury (DILI) in relation to oral doses and blood concentrations. Arch Toxicol 93:1609–1637. https://doi.org/10.1007/s00204-019-02492-9

Fisher C, Siméon S, Jamei M, et al (2019) VIVD: Virtual in vitro distribution model for the mechanistic prediction of intracellular concentrations of chemicals in in vitro toxicity assays. Toxicol In Vitro 58:42–50. https://doi.org/10.1016/j.tiv.2018.12.017

Ford KA, Casida JE (2006) Chloropyridinyl Neonicotinoid Insecticides:  Diverse Molecular Substituents Contribute to Facile Metabolism in Mice. Chem Res Toxicol 19:944–951. https://doi.org/10.1021/tx0600696

Harada KH, Tanaka K, Sakamoto H, et al (2016) Biological Monitoring of Human Exposure to Neonicotinoids Using Urine Samples, and Neonicotinoid Excretion Kinetics. PLOS ONE 11:e0146335. https://doi.org/10.1371/journal.pone.0146335

Kisitu J, Hollert H, Fisher C, Leist M (2020) Chemical concentrations in cell culture compartments (C5) – free concentrations. ALTEX - Altern Anim Exp 37:693–708. https://doi.org/10.14573/altex.2008251

Rodgers T, Rowland M (2007) Rodgers T, Rowland M. 2006. Physiologically‐based Pharmacokinetic Modeling 2: Predicting the tissue distribution of acids, very weak bases, neutrals and zwitterions. J Pharm Sci 95:1238–1257. J Pharm Sci 96:3153–3154. https://doi.org/10.1002/jps.20857

Sugano K (2009) Theoretical investigation of passive intestinal membrane permeability using Monte Carlo method to generate drug-like molecule population. Int J Pharm 373:55–61. https://doi.org/10.1016/j.ijpharm.2009.02.002
